# Regional astrocyte dysregulation and altered glymphatic‐related markers in Alzheimer's disease frontal cortex

**DOI:** 10.1002/alz.71745

**Published:** 2026-08-14

**Authors:** Harry Alexopoulos, Xanthippi P. Louka, Edoardo Rosario De Natale, Kelly Koutroubi, Lisa Cashmore, Maria T. Panayotacopoulou, Ioannis P. Trougakos, Heather Wilson, Marios Politis

**Affiliations:** ^1^ Department of Cell Biology and Biophysics Faculty of Biology National and Kapodistrian University of Athens Athens Greece; ^2^ Neurodegeneration Imaging Group University of Exeter Medical School London UK; ^3^ Department of Psychiatry, Faculty of Medicine National and Kapodistrian University of Athens Athens Greece; ^4^ Department of Neurology, Faculty of Medicine National and Kapodistrian University of Athens Athens Greece

**Keywords:** Alzheimer's disease, amyloid‐beta, aquaporin‐4, astrocyte dysregulation, frontal cortex, glial fibrillary acidic protein, glymphatic system, tau proteins

## Abstract

**INTRODUCTION:**

Astrocyte dysfunction is central to Alzheimer's disease (AD), yet expression patterns of astrocytic markers remain poorly defined. We measured Aquaporin‐4 (AQP4) and glial fibrillary acidic protein (GFAP) in *post‐mortem* frontal cortex of AD patients and controls across BrainNet Europe (BNE) stages.

**METHODS:**

We assessed marker expression across gray and white matter with immunohistochemistry and immunofluorescence.

**RESULTS:**

In AD, gray‐matter AQP4 area–fraction did not differ significantly overall by immunohistochemistry, while a stage‐dependent increase emerged by BNE VI in both gray and white matter. AQP4/amyloid‐β (Aβ) and AQP4/tau ratios were significantly reduced, consistent with reduced AQP4 retention relative to local proteinopathy burden. GFAP intensity was significantly decreased in both gray and white matter of AD patients, with disorganized peri‐plaque morphology in gray matter.

**DISCUSSION:**

These findings reveal compartment‐ and stage‐specific astrocytic dysregulation in AD frontal cortex and identify local loss of AQP4 around proteinopathy. They support investigation of astrocyte/glymphatic‐related pathways as biomarkers and therapeutic targets.

## Highlights

- Global gray‐matter aquaporin‐4 (AQP4) shows no significant differences in Alzheimer's disease (AD), but is significantly reduced locally around amyloid plaques (*p* = 0.026).
- Glial fibrillary acidic protein (GFAP) intensity is significantly decreased in both gray (*p* = 0.008) and white matter (*p* = 0.004) of AD frontal cortex, with disorganised peri‐plaque morphology despite preserved area fraction.
- AQP4 expression rises in late‐stage disease (BrainNet Europe [BNE] VI) in both gray (*p* = 0.003) and white matter (*p* = 0.028), without recovery of plaque‐adjacent AQP4.
- Reduced AQP4/amyloid‐β (Aβ) (*p* = 0.034) and AQP4/tau (*p* = 0.001) ratios indicate failure of AQP4 retention at sites of proteinopathy rather than absolute protein loss.
- Findings support a model of compartment‐ and stage‐specific astrocytic dysregulation in human AD frontal cortex, refining targets for astrocyte‐ and glymphatic‐related biomarker and therapeutic strategies.

## 1 BACKGROUND

Alzheimer's disease (AD) is a progressive neurodegenerative disorder characterized by the accumulation of amyloid‐β (Aβ) plaques and tau neurofibrillary tangles that lead to synaptic loss and neuronal degeneration. While mechanisms of protein aggregation are well established, accumulating evidence shows that non‐neuronal cells and brain clearance systems play critical roles in disease development. Astrocytes, the most abundant glial cells in the brain, and the recently described glymphatic system are increasingly recognized as key contributors to AD pathophysiology.

The glymphatic system facilitates cerebrospinal fluid (CSF)–interstitial fluid (ISF) exchange and clearance of metabolic waste from the brain parenchyma. It is particularly active during slow‐wave sleep, when reduced noradrenergic tone expands the interstitial space and enhances CSF–ISF exchange.^1^ The astrocytic water channel aquaporin‐4 (AQP4), concentrated in astrocytic end‐feet surrounding cerebral vessels, is essential for this perivascular clearance process.^2^

The glymphatic system appears to be compromised in AD,^3^ impairing the removal of toxic proteins like Aβ and tau.^4^ In mice, deletion of the *Aqp4* gene slows perivascular CSF influx into brain tissue and interstitial Aβ clearance, and hastens Aβ plaque formation.^5^, ^6^, ^7^ In a study of *post‐mortem* human frontal cortical tissue from patients with a clinical diagnosis of AD, changes in astroglial AQP4 localization and expression were observed, including the loss of AQP4 from perivascular endfeet and AQP4 redistribution to non‐perivascular processes.^8^ In another human *post‐mortem* case series, including subjects over 65 years of age that were either cognitively intact or had mild cognitive impairment (MCI) or AD, it was observed that reduced frontal cortical perivascular AQP4 localization was associated with local Aβ and phosphorylated tau (p‐tau) pathology in AD and MCI subjects compared to controls.^9^ Additionally, *Aqp4* genetic variation corresponding to glymphatic system functionality is linked to Aβ accumulation and cognitive decline ^10^ and decreased AQP4 CSF levels in AD is an emerging biomarker of disease progression.^11^

The intermediate filament protein glial fibrillary acidic protein (GFAP) is another key astrocytic marker in AD. Under physiological conditions, gray‐matter astrocytes show low GFAP expression. In AD, astrocytes become hypertrophic and strongly GFAP‐positive, particularly around Aβ plaques, reflecting cytoskeletal remodeling and reactive gliosis.^12^ Elevated CSF and plasma GFAP levels correlate with Aβ pathology and cognitive decline,^13^ underscoring its value as both a marker and a potential mediator of astrocytic reactivity.

Despite these insights, the spatial and temporal organization of astrocytic changes in human cortex remains poorly characterized. Specifically, it is unclear how AQP4 and GFAP expression differ between gray and white matter, how these changes evolve with BrainNet Europe (BNE) staging, and how they relate to local Aβ and tau pathology.

We addressed this gap through quantitative histological and immunofluorescent analysis of *post‐mortem* human frontal cortex, integrating whole‐slide imaging with compartment‐specific image quantification. We hypothesized that AQP4 and GFAP expression would show region‐ and stage‐dependent alterations in AD and that local AQP4/GFAP patterns would relate to Aβ and tau pathology. Understanding these compartment‐specific patterns may refine models of astrocyte involvement in AD progression and guide future studies of glymphatic‐related mechanisms.

## 2 METHODS

### 2.1 Ethics statement

All human tissue procedures complied with the Declaration of Helsinki (2013 revision). The study protocol was approved by the local Research Ethics Committee of the National and Kapodistrian University of Athens under approval number: 91245/25.9.2023. All data were managed in accordance with General Data Protection Regulation (GDPR) and the UK Data Protection Act (2018).

### 2.2 Human tissue samples

Tissue samples and associated clinical and neuropathological data were supplied by Parkinson's UK Brain Bank at Imperial, funded by Parkinson's UK, a charity registered in England and Wales (258197) and in Scotland (SC037554). *Post‐mortem* human brain tissue was, also, obtained from the London Neurodegenerative Diseases Brain Bank at King's College London, which receives partial funding as a part of the Brains for Dementia Research program, jointly funded by Alzheimer's Research UK and the Alzheimer's Society. For each subject, a detailed pathology report, including *post‐mortem* intervals (average PMI 39.83 hours for patients and 26.34 hours for controls) was provided by the respective Brain Bank. A total of 54 human brain tissue cases were included in this study, comprising 26 AD patients and 28 aged‐matched controls. Controls were further stratified into two cohorts based on Aβ pathology: controls Αβ‐negative [Αβ(‐), *n* = 13] and controls Αβ‐positive (Αβ(+), *n* = 12). Control Aβ status was available for 25 of 28 controls, because Aβ‐stained sections were not quantifiable in three controls (Table S1). Detailed demographic and clinical characteristics of the subjects are presented in Table 1.

**RESEARCH IN CONTEXT**

1. **Systematic review**: The authors searched PubMed and Google Scholar. Astrocytic dysfunction and glymphatic impairment are contributors to Alzheimer's disease (AD), but the regional and stage‐dependent expression of aquaporin‐4 (AQP4) and glial fibrillary acidic protein (GFAP) in human frontal cortex, in relation to amyloid‐β and tau pathology, remained poorly defined.
2. **Interpretation**: This study demonstrates compartment‐ and stage‐specific astrocytic dysregulation in AD frontal cortex. Gray‐matter AQP4 is largely preserved and rises at late stage (BrainNet Europe [BNE] VI), yet is locally reduced around amyloid plaques. AQP4/Aβ and AQP4/tau ratios were significantly reduced. GFAP falls in gray and white matter, and peri‐plaque morphology is disorganised despite preserved area fraction. These data suggest that AQP4 changes in AD reflect a pathology‐adjacent reduction rather than generalized loss of protein, paralleled by a maladaptive astrocytic reactivity profile.
3. **Future directions**: Future work should combine AQP4 with vascular markers to quantify perivascular/parenchymal ratios, distinguishing astrocyte subtypes, and refining targets for astrocyte‐ and glymphatic‐related biomarkers and therapeutics.

**TABLE 1:** Summary of demographic and neuropathological characteristics of AD patients and controls.

AD patients							Controls						
ID	Sex		Age	AD BNE stage		PMI	ID	Sex		Age	PMI	Aβ42 status	
AD1	M		88	III–IV	*N* = 9 Age (mean) = 89.56	79	C1	M		58	9	(−)	
AD2	M		78	IV		36	C2	F		61	15	(−)	
AD3	M		86	IV		52.5	C3	F		71	17		
AD4	F		86	IV		55.5	C4	M		74	72		
AD5	M		90	IV		38	C5	M		75	12	(−)	
AD6	M		91	IV		48	C6	M		79	25	(−)	
AD7	F		100	IV		18	C7	F		82	20	(+)	
AD8	M		92	IV		65	C8	F		82	15	(−)	
AD9	F		95	IV		47	C9	M		83	26	(+)	
AD10	M		75	V	*N* = 9 Age (mean) = 87.11	36	C10	M		84	32	(+)	
AD11	M		84	V		29	C11	M		85	29	(+)	
AD12	M		88	V		33	C12	M		87	17	(−)	
AD13	F		88	V		67	C13	M		87	48	(+)	
AD14	F		92	V		25	C14	M		88	8	(+)	
AD15	M		93	V		44	C15	M		89	21	(−)	
AD16	M		94	V		24	C16	M		89	18	(−)	
AD17	F		89	V		59	C17	F		89	22	(+)	
AD18	F		81	V		49	C18	F		89	13	(−)	
AD19	F		92	VI	*N* = 8 Age (mean) = 82	27	C19	M		90	12	(−)	
AD20	M		81	VI		30	C20	F		91	51	(+)	
AD21	F		89	VI		8.5	C21	F		92	63	(−)	
AD22	M		75	**VI**		78	C22	F		92	24	(+)	
AD23	M		74	VI		31	C23	F		93	46	(−)	
AD24	F		85	VI		12	C24	F		94	40	(+)	
AD25	F		86	VI		25	C25	F		95	28	(+)	
AD26	F		74	VI		19	C26	M		95	11	(+)	
							C27	M		97	19.5	(−)	
							C28	F		98	24		
AD patients	M	14	Mean			Mean	Controls	M	15	Mean	Mean	(−)	13
	F	12	86.38			39.83		F	13	85.32	26.34	(+)	12

*Note*: The cohort consisted of 26 AD patients and 28 control subjects. AD patients are categorized by their neuropathological stage according to BNE criteria. Controls are stratified based on their amyloid‐β status [(‐) or (+)].

Abbreviations: AD: Alzheimer's disease; BNE: BrainNet Europe; F: female; M: male; PMI: *post‐mortem* interval.

### 2.3 Immunohistochemistry staining of brain frontal cortex tissue sections

Frontal cortex 7‐µm‐thick formalin‐fixed paraffin‐embedded (FFPE) serial tissue sections were deparaffinized and rehydrated using successive rinsings in xylene and ethanol. Serial sectioning ensures that any staining observations are evaluated within the same anatomical microenvironment and across identical cortical columns. For antigen retrieval and blocking, we used the heat‐induced method in citrate buffer (pH = 6) and 5% bovine serum albumin, respectively. After washing with phosphate‐buffered saline with Triton‐X 0.025% (PBS‐T), slices were incubated overnight at 4°C with primary antibodies. The primary antibodies used in this part of the study were: rabbit polyclonal anti‐AQP4 antibody (Novus, Cat# NBP1‐87679, 1:1500), rabbit polyclonal anti‐GFAP antibody (Novus, Cat# NBP2‐33774, 1:1500), and rabbit monoclonal anti‐Aβ antibody (Cell Signaling, Cat# D54D2 ‐ 8243, 1:1000). Subsequently, 0.3% H_2_O_2_ in PBS was used for quenching endogenous peroxidase activity for 15 minutes at room temperature. For staining detection, we used the VECTASTAIN Elite ABC system (Vector Laboratories, Cat# PK‐6101). The sections were incubated with the secondary antibody (Goat anti‐rabbit, Vector, Cat# PK‐6101, 1:1500) for 30 minutes at room temperature. Peroxidase enzymatic activity was visualized using diaminobenzidine (DAB) as a substrate, following enhancement steps. Sections were then counter‐stained with hematoxylin (Gill No. 3, Sigma‐Aldrich) for nuclei labeling, dehydrated, and mounted in a xylene‐based mounting medium. Whole slides were scanned at 20× magnification using the PALM MicroLaser System (Carl Zeiss).

### 2.4 Immunofluorescence staining of brain frontal cortex tissue sections

For immunofluorescence (IF), FFPE tissue sections from human frontal cortex were initially processed as in the immunohistochemistry (IHC) protocol until the incubation step with the first antibody. The same primary antibodies were used for AQP4 and GFAP at the same dilutions, and the mouse monoclonal anti‐tau antibody (Abcam, Cat# ab80579, 1:1000,) for tau. The tissue sections were incubated with the secondary antibody (1:2000) for 60 minutes at room temperature in the dark. The secondary antibodies used were goat anti‐rabbit IgG 488 (Biotium, Cat# 20012) and goat anti‐mouse IgG 568 (Biotium, Cat# 20101). For protein aggregation detection, tissues were incubated for 30 minutes with the reagent Amytracker 680 (Ebba Biotech, Cat# 4‐3A‐A680) at dilution 1:1000 after the secondary antibody. For mounting, the VECTASHIELD HardSet™ Antifade Mounting Medium with DAPI (Vector, Cat# H‐1500) was used. Samples were imaged using a Digital Eclipse Nikon C1 confocal laser scanning microscope (CLSM) (Nikon Corporation, Tokyo, Japan). All images were acquired under identical exposure and gain settings to ensure comparability, with consistent laser power and detector gain applied to minimize variability and ensure reliable fluorescence quantification.

### 2.5 Image analysis

For [Fig alz71745-fig-0001]IHC analysis, as mentioned above, the whole tissue slide was scanned at magnification 20×. From this procedure, approximately 900–1500 individual images were generated for each slide/subject. Then, using the QuPath Software (v0.5.1) for bioimage analysis,^14^ we manually annotated the whole‐slide composite image by drawing specific rectangular areas in the gray (red boxes) and white (blue boxes) matter, respectively. Following, to automate the selection and categorization of individual images corresponding to these manually annotated regions of interests (ROIs), a custom Python script was developed. The script identified the coordinates of the red and blue boxes and sorted the relevant individual images into separate folders based on their spatial location within the annotated regions.

A custom Python (v3.10)‐based pipeline was developed to quantify DAB‐immuno‐stained tissue regions across the extracted individual images from gray and white matter separately. Images were processed using the OpenCV (v4.8), NumPy (v1.24), pandas (v2.0), and scikit‐image (v0.21) libraries.

Red–green–blue (RGB) images were converted to optical density (OD) space using the Beer‐Lambert law (OD = —log_10_(I/I_0_)), which establishes a linear relationship between chromogen concentration and light absorbance. DAB and hematoxylin chromogens were separated via linear unmixing using custom stain‐specific vectors generated manually using QuPath. To correct for non‐specific DAB background, we characterized technical background using respective selected images in which no specific immunoreactivity is expected. Then, DAB‐positive pixels were identified using an adaptive threshold method combining global background statistics with local tissue characteristics. This approach maintains sensitivity and consistency across samples with varying staining intensities.

Two primary metrics were calculated for each image. Area percentage (%), defined as the proportion of tissue pixels exceeding the detection threshold, reflecting the spatial extent of protein expression. This metric is normalized to tissue area to account for variations in tissue integrity. The second metric, mean DAB intensity, was calculated as the average optical density of threshold‐positive pixels, representing average protein concentration in expressing regions. The analysis was applied batch‐wise across individual subject folders with automated generation of binary visualization masks for manual quality control. Quantitative results were exported to structured comma‐separated values (CSV) files for downstream statistical analysis. Subject‐level values were calculated as tissue‐weighted averages across all analyzed tiles to account for varying tile numbers between subjects.

For immunofluorescence analysis, whole‐slide tissue sections were manually examined across three fluorescent channels and high‐magnification images (20× objective) were acquired focusing on protein aggregate deposits in gray matter. Due to limitations, for example, lipofuscin autofluorescence and signal attenuation from photobleaching, the analysis was performed in a semi‐quantitative manner. To minimize the impact of non‐specific background signals on our findings, we utilized integrated density ratios rather than absolute fluorescence values. By calculating these ratios within the same ROIs, the measurements are internally normalized. Image processing and quantification were carried out using Fiji (v6.5).^15^ Each three‐channel image was spilt into individual color channels. Circular ROIs (width: 200 pixels, height: 200 pixels) were manually placed over protein aggregate deposits identified in the red channel (Amytracker 680). Then, for each ROI, the integrated density was measured in both the red (aggregates) and green (AQP4 or GFAP) channels. Integrated density in each ROI reflects both deposit size and fluorescence intensity. Signal stability across samples was confirmed by consistent exposure settings and comparable background levels. For each ROI, the expression of AQP4 or GFAP in relation to protein aggregates was estimated by calculating the ratio of integrated density in the green channel to that in the red channel. The final metric per subject was obtained by averaging this ratio across all ROIs analyzed.

To facilitate reproducibility and transparent reporting of computational methods, all Python scripts developed for image analysis in this study are publicly available on GitHub (see the Data and Code Availability section).

### 2.6 Statistical analysis

Data[Fig alz71745-fig-0002], [Fig alz71745-fig-0003] were collected using Microsoft Excel and analyzed and visualized using GraphPad Prism (v.8; GraphPad Software, San Diego, CA, USA). To ensure statistical robustness, all datasets were subjected to a pre‐specified formal outlier identification test using the ROUT method (*Q* = 1%) in GraphPad Prism. Cases identified as statistical outliers were excluded from the respective analysis. Consequently, the total number of samples (*n*) may vary slightly between individual metrics and tissue regions; these variations are noted in the respective figure legends. Firstly, data was assessed for normality using the Shapiro–Wilk test. Depending on the data distribution, comparisons between two groups were conducted using either the parametric unpaired two‐tailed Student's *t*‐test or the non‐parametric unpaired Mann–Whitney *U* test. For multiple group comparisons, one‐way analysis of variance (ANOVA) followed by Tukey's post hoc test (parametric) or Kruskal–Wallis followed by Dunn's test (non‐parametric) was applied. Group differences in astroglial markers were assessed using multiple linear regression with DAB metrics (AREA (%), Mean DAB Intensity) as the dependent variable and disease group (controls vs. AD patients, Αβ‐ vs. Αβ+ subjects), age, sex, and PMI as independent variables. This approach controls confounding effects of demographic and tissue quality variables. The analysis was performed using the ordinary least squares (OLS) method according to the following general equation: Y = β_0_ + β_1_*A (Group) + β_2_*B (Sex) + β_3_*C (Age) + β_4_*D (PMI), where Y is the dependent variable and β_0_ the intercept. Group and Sex were treated as categorical variables. The model was fitted using an unweighted approach, assuming equal variance across all observations. Model assumptions, including normality of residuals and homoscedasticity, were verified through residual diagnostics. Coefficient estimates (β) and 95% confidence intervals (CI) were calculated with statistical significance defined as *p*‐value < 0.05. Full statistical output for all linear models—including coefficient estimates (β), standard errors, 95% CIs, t‐statistics, and *p*‐values for each term, together with model‐level *R* ^2^ and F‐statistics—is provided in Table S2.

## 3 RESULTS

**FIGURE 1 alz71745-fig-0001:**
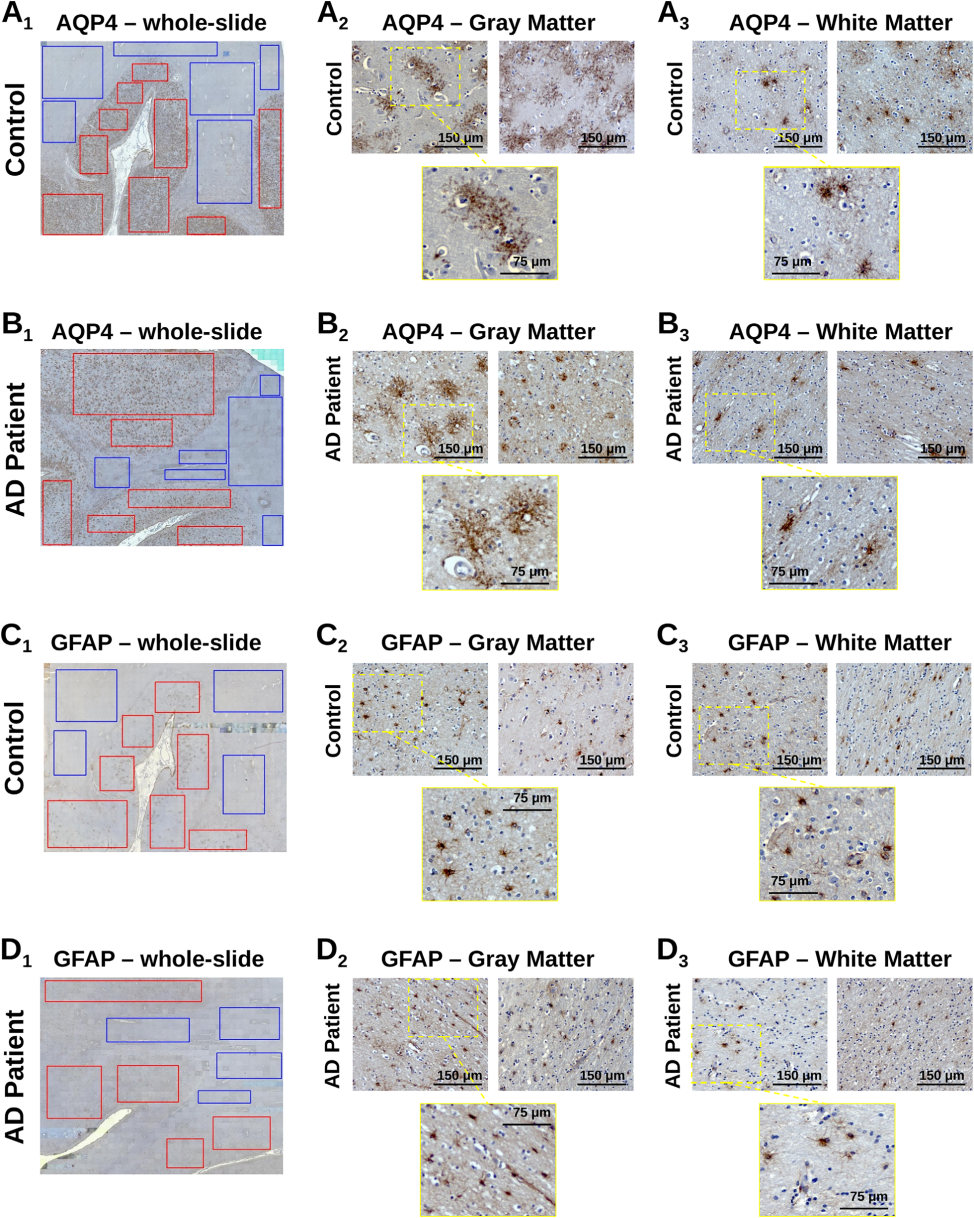
AQP4 and GFAP staining pattern in the frontal cortex of AD patients. (A, B) Representative whole slide images of immunohistochemical staining for AQP4 in human brain frontal cortex sections from a control sample (A_1_) and a patient sample (B_1_). Representative higher magnification (20× objective) immunohistochemical images of AQP4 expression in the gray matter of a control (A_2_) and a patient (B_2_) sample. Representative immunohistochemical images (20× magnification) of AQP4 expression in the white matter of a control (A_3_) and a patient (B_3_) sample. (C, D) Representative whole slide images of immunohistochemical staining for GFAP in human brain frontal cortex sections from a control sample (C_1_) and a patient sample (D_1_). Representative immunohistochemical images (20× magnification) of GFAP expression in the gray matter of a control (C_2_) and a patient (D_2_) sample. Representative immunohistochemical images (20× magnification) of GFAP expression in the white matter of a control (C_3_) and a patient (D_3_) sample. The blue and red boxes, designed using QuPath image processing software, indicate different ROIs in the white and gray matter, respectively. Yellow dashed boxes indicate ROIs shown as high‐magnification insets below. Brown staining indicates AQP4‐ and GFAP‐positive regions, respectively. The scale bar is equal to 150 µm (main images) and 75 µm (insets). AD, Alzheimer's disease; AQP4, aquaporin‐4; GFAP, glial fibrillary acidic protein; ROI, region of interest.

Our primary hypothesis, that AQP4 and GFAP expression would show region‐ and stage‐dependent alterations in AD and that local AQP4/GFAP patterns would relate to Aβ and tau pathology was investigated in human frontal cortex. Peroxidase‐based immunohistochemistry was performed on frontal cortex tissue sections from AD patients and controls, with gray and white matter analyzed separately as distinct compartments. To accurately quantify protein expression, we implemented a digital pathology pipeline involving color deconvolution, followed by automated thresholding to generate binary masks (Suppl. Figure 1). Representative whole‐slide sections stained for AQP4 and GFAP, respectively, from controls and AD patients are shown in Supplementary Figure 2. From these sections, rectangular ROIs were selected from white (blue boxes) and gray (red boxes) matter, respectively (Figure 1A_1_, B_1_, C_1_, D_1_). High magnification images from these ROIs along with smaller sub‐regions (yellow boxes), being representative of the corresponding staining patterns in controls (Figure 1A_2_, A_3_, C_2_, C_3_) and patients (Figure 1B_2_, B_3_, D_2_, D_3_) are also shown.

### 3.1 Immunohistochemical analysis: Global changes

Prior to investigating the global changes in glymphatic system‐related proteins, we characterized the Αβ deposition in our cohort subjects. Protein levels were assessed using two distinct metrics: Area (%), representing the total parenchymal coverage, and Mean DAB Intensity (OD), representing the relative protein concentration per pixel. We observed that Aβ aggregates were only presentin the gray matter, as expected. Therefore, we manually selected ROIs only in gray matter (red boxes in Suppl. Figure 3A_1_, B_1_, and C_1_). The AD group showed significantly increased Aβ accumulation compared to the control group by both metrics (Area (%) *p*‐value < 0.0001, Mean DAB Intensity *p*‐value = 0.0001) (Suppl. Figure 4A_2_, B_2_). When testing AD patients by BNE stage, no statistically significant differences in Aβ burden were observed across stages (Suppl. Figure 4A_3_, B_3_). It is important to point out, that our control cohort included Aβ+ subjects, as shown in the representative images in Supplementary Figure 3A_2_ and B_2_. Therefore, we did an analysis where the control cohort was divided into two groups based on the absence (control Αβ‐) or the presence (control Αβ+) of amyloid pathology.

**FIGURE 2 alz71745-fig-0002:**
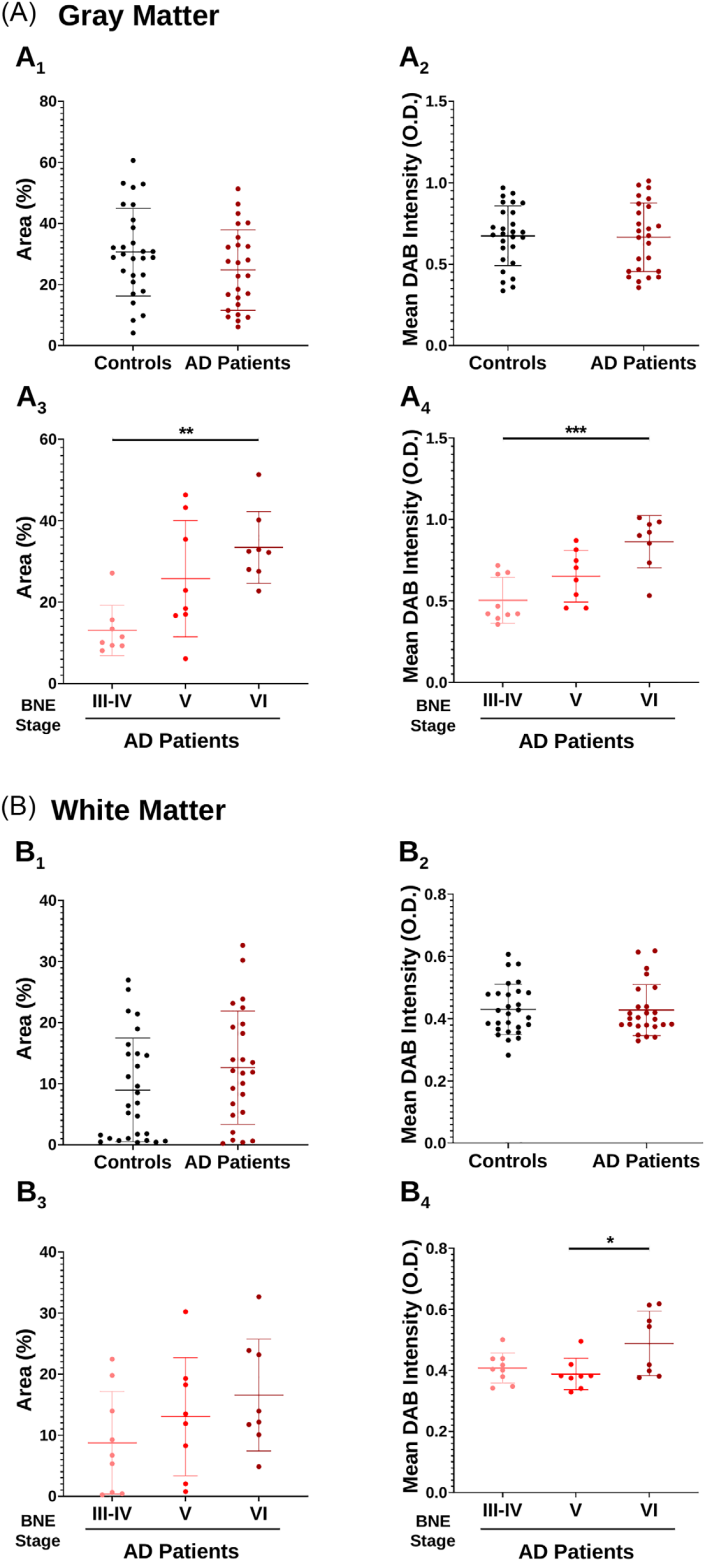
Quantification of AQP4 expression in the frontal cortex of AD patients. (A) Analysis of gray‐matter images. (A_1_, A_2_) The average value of the AQP4 Area (%) and Mean DAB Intensity (OD) between controls and AD patients. Number of values: N_Controls _= 28, N_Patients _= 25 for Area (%) and N_Controls _= 27, N_Patients _= 25 for Mean DAB Intensity. (A_3_, A_4_) The average value of the above AQP4 metrics for each patient stratified by BNE stage (III–IV, V, and VI). Number of values: N_III‐IV _= 8, N_V _= 8, and N_VI _= 8 for Area and N_III‐IV _= 9, N_V _= 8, and N_VI _= 8 for Intensity. (B) Analysis of white‐matter images. (B_1_, B_2_) The average of AQP4 Area (%) and DAB Intensity in each subject of the control and AD patient cohort. Number of values: N_Controls _= 28, N_Patients _= 25 for both metrics. (B_3_, B_4_) The mean value of AQP4 metrics for each patient grouped by BNE stage (III–IV, V, and VI). Number of values: N_III‐IV _= 9, N_V _= 8, and N_VI _= 8 for both metrics. Asterisks indicate statistically significant differences: *, *p*‐value < 0.05; **, *p*‐value < 0.01; ***, *p*‐value < 0.001. AD, Alzheimer's disease; AQP4, aquaporin‐4; BNE, BrainNet Europe; DAB, diaminobenzidine.

Next, we quantified AQP4 expression in AD tissue compared to controls. The distribution of individual data points from every captured image across all studied groups is presented for both metrics in Supplementary Figures 5A, 6A. In the gray matter, we found that AQP4 Area (%) did not differ significantly between AD patients and controls (Figure 2A1), which was also reflected in the amyloid‐positive control cohort (Suppl. Figure 7A_1_). To determine if any clinical characteristics predicted this metric, we performed multiple linear regression. For both the global comparison (controls vs. AD patients) and the amyloid‐status comparison (Αβ‐ vs. Αβ+), no variables (Group, Age, Sex, PMI) emerged as significant independent predictors of this AQP4 metric (Suppl. Figure 7C_1, 2_). Univariate analysis, likewise, showed no significant difference in AQP4 Mean DAB Intensity between controls and patients (Figure 2A2), even when sub‐categorizing controls by amyloid status (Suppl. Figure 7A_2_). However, multiple linear regression identified PMI as a significant independent predictor of AQP4 intensity (*p*‐value = 0.013 for global comparison, *p*‐value = 0.030 for amyloid status comparison). While AD patients exhibited a higher average PMI, the diseased group remained a non‐significant factor after statistical adjustment, confirming that AQP4 protein concentration in gray matter is largely preserved across our cohort.

In the white matter, AQP4 Area (%) did not differ significantly between AD patients and Aβ+ controls compared to Αβ‐ controls (Figure 2B1, Suppl. Figure 7B_1_). Multivariate regression analysis confirmed a non‐significant group effect. Notably, sex was identified as a significant independent predictor of white matter AQP4 Area (%) (*p*‐value = 0.035) in the amyloid‐stratified model, suggesting that biological sex contributes more significantly to the variance in white‐matter coverage than amyloid status alone.

Next, we examined if there was any difference in AQP4 expression in AD patients according to BNE stages (Suppl. Figures 5B, 6B). In the gray matter, we observed a progressive, stage‐dependent increase in AQP4 expression. Specifically, the patients in BNE stage VI exhibited higher AQP4 Area (%) and Mean DAB Intensity levels compared to BNE stage III–IV patients (*p*‐value = 0.003 and *p*‐value = 0.001, respectively) (Figure 2A3, 4). In the white matter, while Area (%) did not differ significantly across stages (Figure 2B3), we observed a statistically significant increase in AQP4 expression levels in BNE stage VI AD patients compared to BNE stage V patients (*p*‐value = 0.028) (Figure 2B4).

**FIGURE 3 alz71745-fig-0003:**
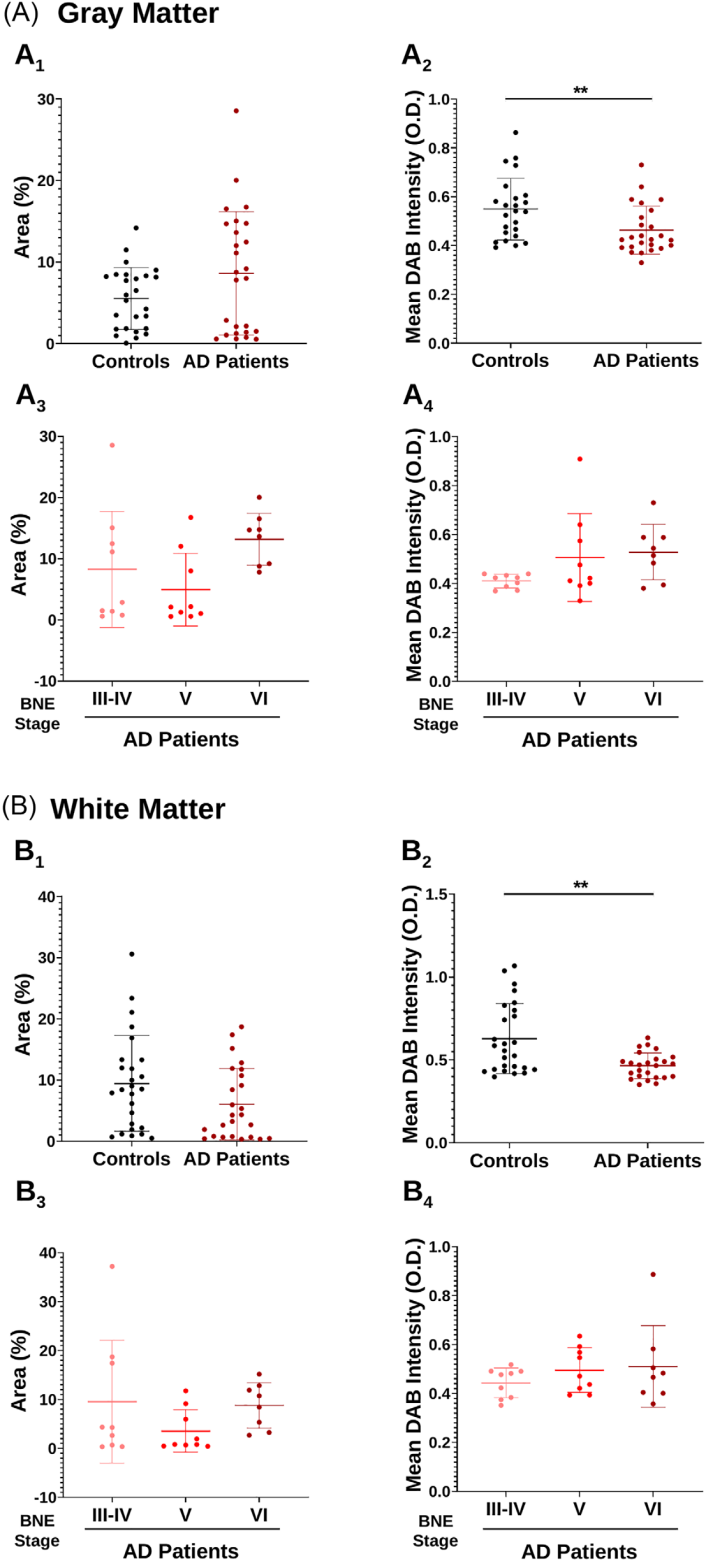
Quantification of GFAP expression in the frontal cortex of AD patients. (A) Analysis of gray‐matter images. In (A_1_, A_2_) the average values of the GFAP Area (%) and Mean DAB Intensity (OD) are presented between controls and AD patients. Number of values: N_Controls _= 26, N_Patients _= 26 for Area (%) and N_Controls _= 24, N_Patients _= 25 for Mean DAB Intensity. (A_3_, A_4_) represent the average value of the above GFAP metrics for each patient stratified by BNE stage (III–IV, V, and VI). Number of values: N_III‐IV _= 9, N_V _= 9, and N_VI _= 8 for both metrics. (B) Analysis of white‐matter images. (B_1_, B_2_) The average of GFAP Area (%) and DAB Intensity in each subject of the control and AD patient cohort. Number of values: N_Controls _= 26, N_Patients _= 25 for both metrics. (B_3_, B_4_) represent the mean value of GFAP metrics for each patient grouped by BNE stage (III–IV, V, and VI). Number of values: N_III‐IV _= 9, N_V _= 9, and N_VI _= 8 for both metrics. Asterisks indicate statistically significant differences: **, *p*‐value < 0.01. AD, Alzheimer's disease; BNE, BrainNet Europe; DAB, diaminobenzidine; GFAP, glial fibrillary acidic protein.

Next, we quantified GFAP expression in AD tissue compared to controls across gray and white‐matter compartments. We first visualized the distribution of individual image values across all subjects (Suppl. Figures 8A, 9A). In the gray matter, univariate analysis showed non‐significant difference in GFAP Area (%) between AD patients and controls (Figure 3A1), including when controls were sub‐categorized by amyloid status (Suppl. Figure 10A_1_). Multiple linear regression confirmed that sex is a significant independent predictor of this metric in both studied models (*p*‐value = 0.010 and *p*‐value = 0.014, respectively) (Suppl. Figure 10C_1, 2_). While the disease group showed a positive estimate, the significance of biological sex suggests that GFAP area is not uniform across our cohort but is significantly influenced by sex‐specific factors. However, GFAP expression levels decreased by 21.4% in the gray matter of AD patients (*p*‐value = 0.008) (Figure 3A2), a result that was maintained when comparing AD patients specifically to Αβ‐ controls (*p*‐value = 0.048) (Suppl. Figure 10A_2_). Multiple linear regression confirmed the AD group as a significant independent predictor of reduced GFAP intensity in the global comparison (*p*‐value = 0.029). In the white matter, univariate analysis revealed a significant reduction in GFAP Mean DAB Intensity in AD patients compared to both global controls (*p*‐value = 0.004) (Figure 3B_2_) and Αβ‐ controls (*p*‐value = 0.012) (Suppl. Figure 10B_2_); GFAP Area (%) did not differ significantly between groups (Figure 3B1, Suppl. Figure 10B_1_). Multiple linear regression confirmed the disease group as a significant independent predictor of reduced GFAP intensity in both the global and the amyloid‐deposit models (*p*‐value = 0.003 and *p*‐value = 0.012, respectively). In contrast, sex emerged again as a significant independent predictor of GFAP Area (%) in both regression models (*p*‐value = 0.009 and *p*‐value = 0.012, respectively). Following patient stratification by BNE stage, the GFAP expression in both regional compartments appeared relatively independent of BNE stage (Figures 3A3, 4, B3, 4). Scatter plots reflecting data distribution of GFAP metric values for each patient based on the BNE stage are shown in Supplementary Figures 8B, 9B.

**FIGURE 4:**
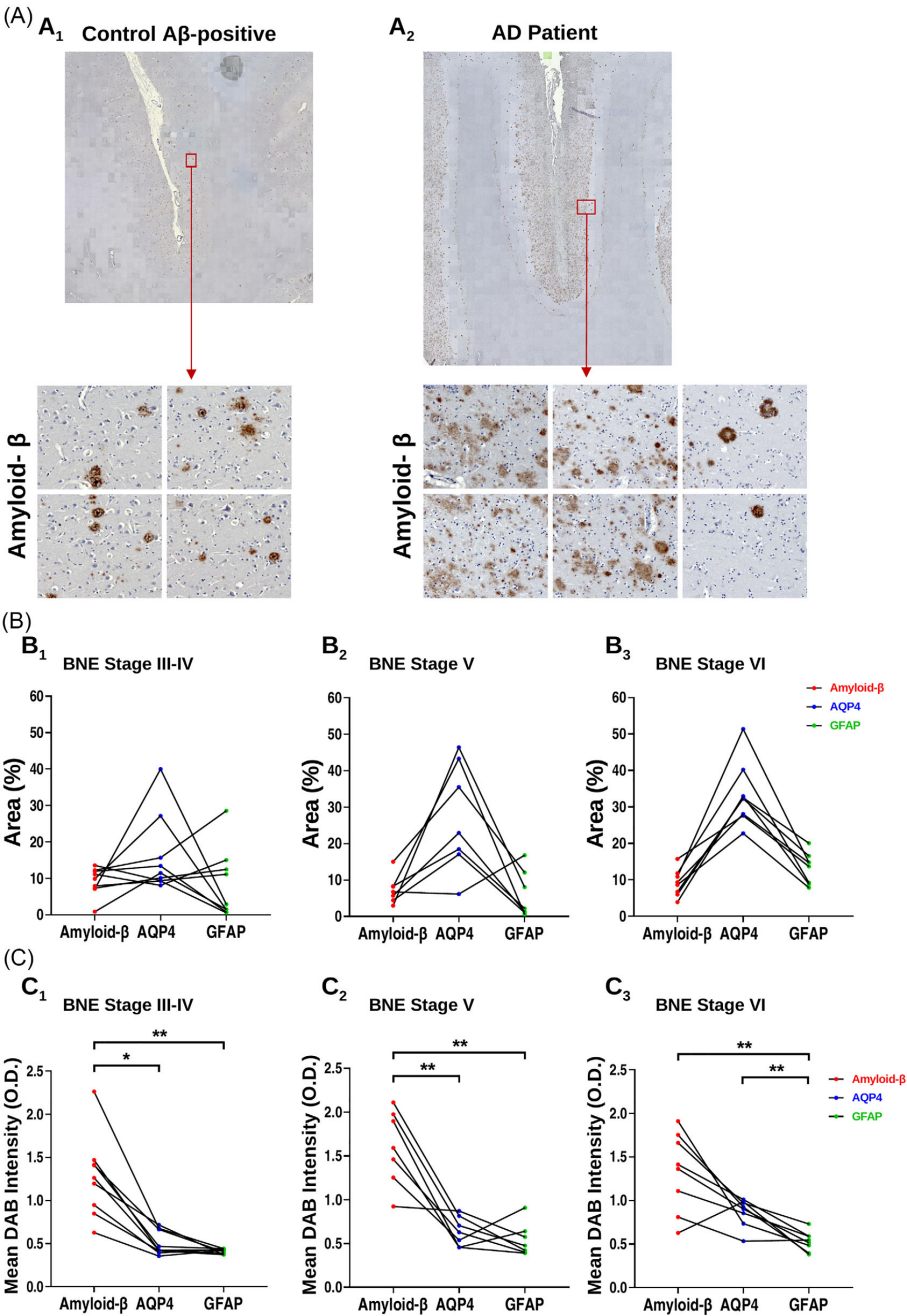
Visualization of amyloid pathology and quantification of associated markers immunoreactivity across BNE stages in the frontal cortex gray matter. (A)_._ Representative whole slide immunohistochemical images with Αβ+ staining from human brain frontal cortex tissue sections from a control Αβ+ sample (A_1_) and a patient sample (A_2_). Red boxes indicate ROIs in the gray matter and the gray–white matter border, while higher magnification (20× objective) immunohistochemical images of these selected ROIs show the distribution of Αβ aggregates across gray matter in the control and the patient sample, respectively. (B) Paired analysis of parenchymal coverage. (B_1_–B_3_) Line plots showing, for each AD patient, the average value of Area (%) for Αβ (red), AQP4 (blue), and GFAP (green), respectively, in gray matter. Each line connects the values obtained from the same patient. The different plots present the AD patients belonging to the same BNE stage; BNE III–IV stage (B_1_), BNE V stage (B_2_), and BNE VI stage (B_3_). (C) Paired analysis of protein concentration. (C_1_–C_3_) Line plots comparing the average value of mean intensity for each marker in gray matter across disease stages. Each line connects the values obtained from the same patient. The different plots present the AD patients belonging to the same BNE stage. Asterisks indicate statistically significant differences: *, *p*‐value < 0.05; **, *p*‐value < 0.01. Aβ, amyloid‐β; AD, Alzheimer's disease; BNE, BrainNet Europe; ROI, region of interest.

Regarding amyloid pathology, our study revealed a divergence in Αβ deposition patterns between groups. In control samples displaying Aβ positivity, Aβ deposition was limited and largely consisted of a few relatively big, well‐defined plaques (Figure 4A1). In contrast, AD samples showed a heterogenous population of Aβ aggregates, including a mix of small oligomeric deposits and larger dense‐core plaques. Furthermore, in the AD samples, focusing away from the gray‐matter toward the gray‐white matter border, a spatial shift from smaller aggregates to larger plaques was observed (Figure 4A2). Notably, a distinct boundary line was apparent, enriched with larger plaques. Further, we examined the distribution of astrocytic markers in corresponding whole‐slide images (Suppl. Figure 11). In both controls and AD patients, we observed that AQP4 is highly enriched throughout the gray matter but terminates at the gray–white matter junction. This boundary seems to be further highlighted by a denser arrangement of GFAP‐positive astrocytes‐most notably in AD subjects‐ forming a continuous “line” along the border. These findings suggest that the specific enrichment of larger plaques in this region may be linked to an anatomical or functional glial barrier that affects amyloid accumulation and plaque maturation.

Following the IHC analysis for each marker, we next aimed to assess the relationship between protein deposition and astrocytic response within individual subjects by performing a paired analysis of Aβ, AQP4, and GFAP in the gray matter per patient disease stages (Figures 4B, C). In the early‐to‐moderate stages (BNE III–IV), Αβ and AQP4 area coverage remained relatively balanced (Figure 4B1). However, as pathology advanced, a notable divergence pattern emerged (Figures 4B2, 3). In contrast, GFAP Area (%) remained consistently lower than AQP4 across all BNE stages. The analysis of protein concentration revealed a consistent hierarchical pattern across all disease stages. Αβ deposits exhibited significantly higher Mean DAB Intensity compared to both AQP4 and GFAP (AQP4 *p*‐value = 0.014, GFAP *p*‐value = 0.001 for III–IV stages, AQP4 *p*‐value = 0.004, GFAP *p*‐value = 0.005 for V stage, GFAP *p*‐value = 0.003 for VI stage) (Figure 4C). In BNE stage VI, while the Αβ intensity remained high, the AQP4 intensity values showed tighter clustering. This suggests that while AQP4 coverage expands in the late‐stage AD, the actual concentration of the protein per pixel remains lower than that of the primary amyloid pathology.

### 3.2 Immunofluorescent analyses: Localized changes

**FIGURE 5:**
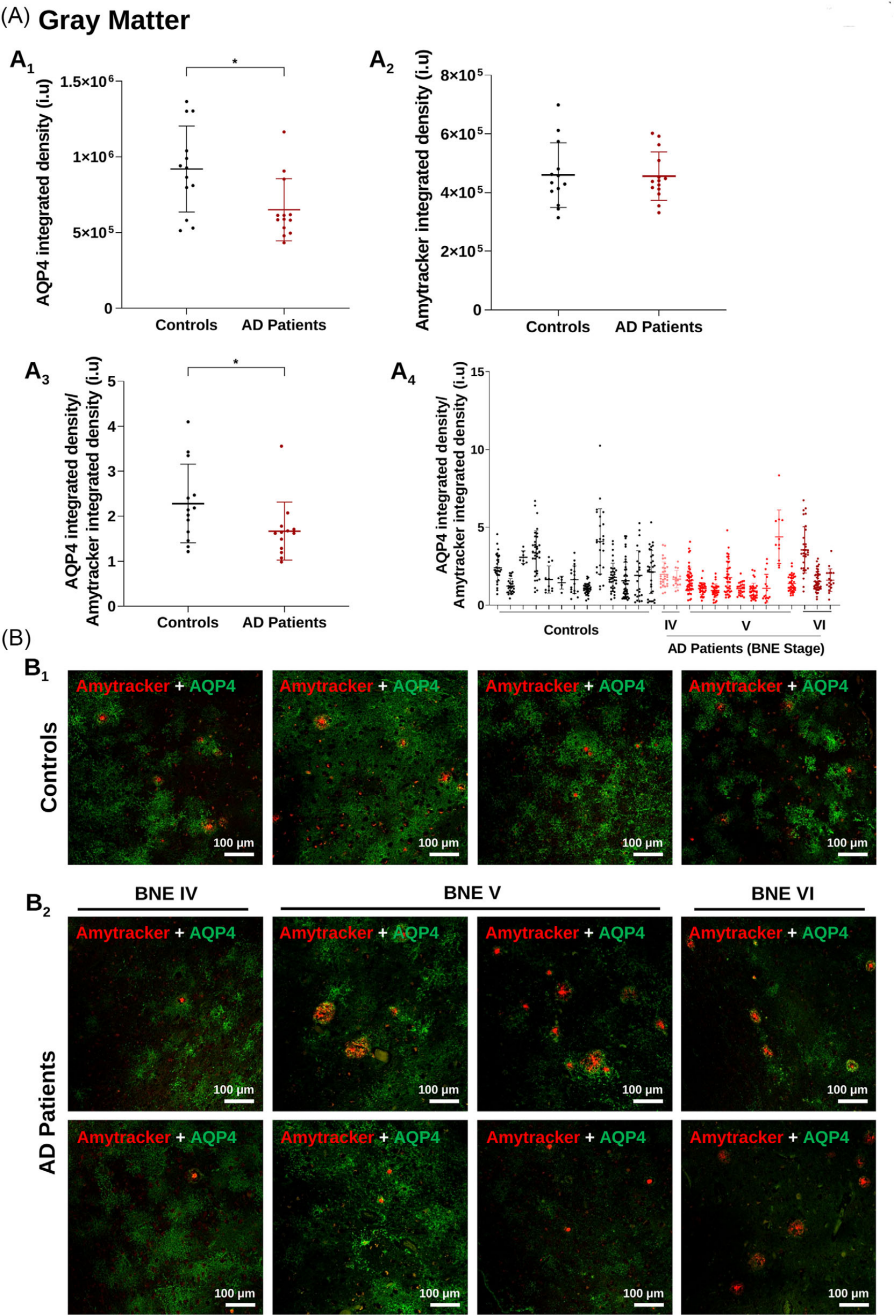
Quantification of AQP4 expression around regions of protein accumulation in the frontal cortex gray matter of AD patients. (A) Quantification of AQP4 and protein aggregates presence in gray‐matter immunofluorescence images of control and patient samples. A_1_ presents the AQP4 integrated density measured in intensity units (iu), a metric used to quantify fluorescence intensity. The scatter plots show the mean value of integrated density measured in the unique ROIs of each subject from the control and patient group. Number of values: N_Controls _= 13 control samples, N_Patients _= 13 patient samples. In A_2_, the scatter plot represents the average value of Amytracker 680 integrated density in the control and patient groups. Number of values: N_Controls _= 13 control samples, N_Patients _= 14 patient samples. In A_3_ the mean value of the AQP4/Amytracker 680 integrated density ratios measured in each subject is presented in a scatter plot. Number of values: N_Controls _= 13 control samples, N_Patients _= 13 patient samples. A_4_ indicates the distribution of AQP4/Amytracker 680 integrated density ratios for the ROIs of individual subjects by further grouping the patient samples based on BNE stage (III–IV, V, and VI). (B) Representative immunofluorescence images of AQP4 (green) and protein aggregates (red, Amytracker 680) in frontal cortex gray‐matter tissue sections. B_1_ shows stained images from control samples. B_2_ shows images from patient samples further categorized by BNE stage (IV, V, and VI). In B, the scale bar is equal to 100 µm. Asterisks indicate statistically significant differences: *, *p*‐value < 0.05. AD, Alzheimer's disease; AQP4, aquaporin‐4; BNE, BrainNet Europe; ROI, region of interest.

Next, we sought to locally measure AQP4 and GFAP expression specifically around regions of protein aggregate deposition. We double stained tissue with AQP4 or GFAP and the Amytracker 680 dye, which labels protein aggregates with repetitive arrangement of β‐sheets, including Αβ plaques and neurofibrillary tangles. We imaged our tissue sections with confocal microscopy. For analysis, we manually selected plaque‐centered ROIs located in the gray matter and measured the integrated density in the selected ROIs in both green (AQP4 or GFAP) and red (Amytracker 680) channels, as shown in Supplementary Figure 12A. Measuring AQP4 density in Aβ+ ROIs, revealed that total AQP4 density was significantly lower in AD patients compared to controls (*p*‐value = 0.026) (Figure 5A1), while the levels of protein aggregates in both groups did not differ (Figure 5A2). However, when we normalized AQP4 intensity to the local Aβ load within our plaque‐associated ROIs, the AQP4 to Aβ density ratio was significantly lower in the patients compared to controls (*p*‐value = 0.034) (Figure 5A3). This shows that locally, around amyloid plaques, astrocytic AQP4 expression is lessened in AD patients. Stratifying the AD patients based on BNE stage revealed that this reduction in local AQP4/Aβ ratio persists across disease stages, without any significant pattern being observed (Figure 5A4). Representative immunofluorescence images illustrate the distribution of AQP4 in the presence of protein pathology (Figure 5B). In control samples, AQP4 exhibited a diffuse pattern, with low Amytracker‐positive signal reflecting low deposit burden. In contrast, AD patient samples across BNE stages showed increased Aβ plaque density, accompanied by reduced AQP4 signal within Amytracker‐positive plaque‐associated ROIs.

**FIGURE 6:**
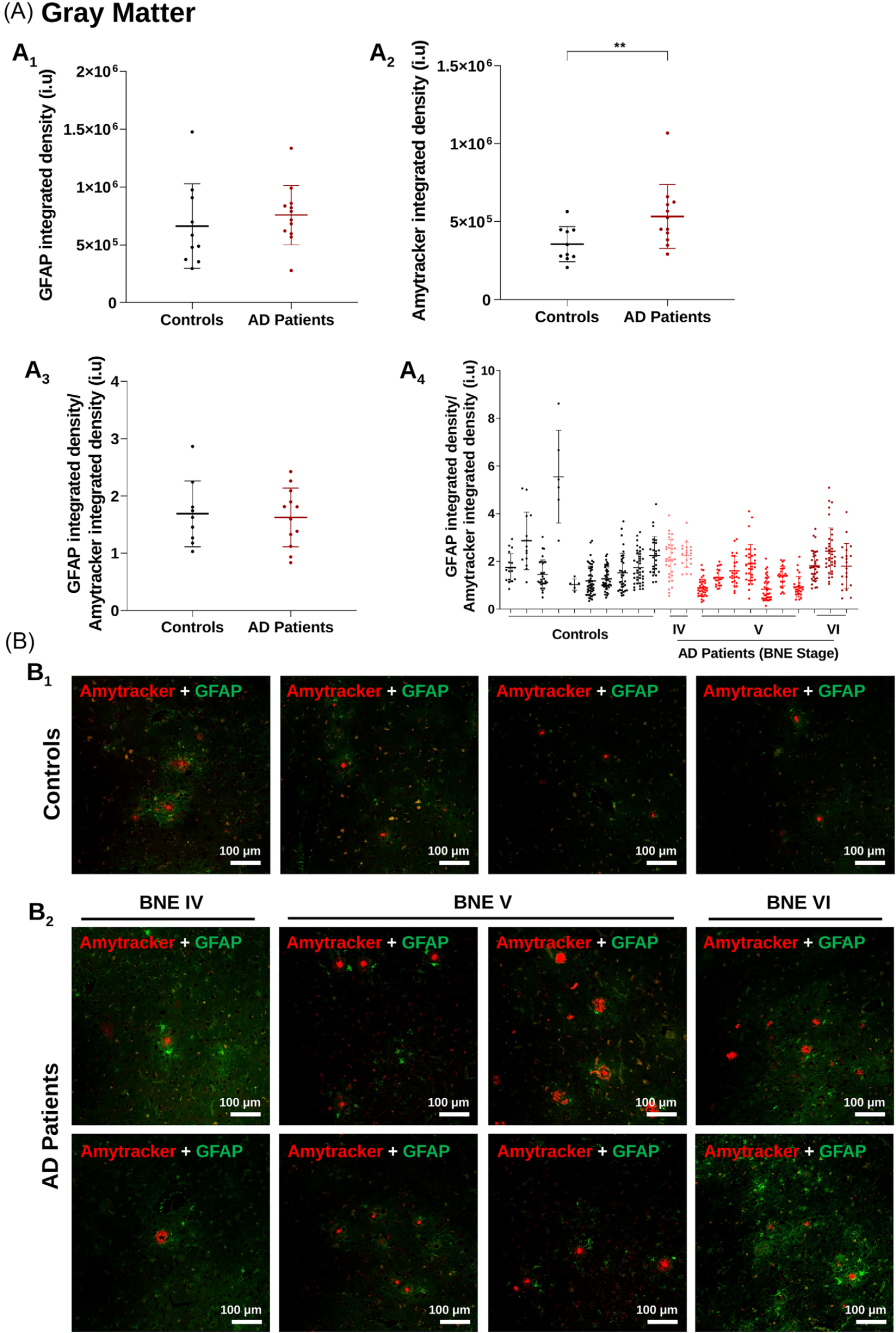
Quantification of GFAP expression around regions of protein accumulation in the frontal cortex gray matter of AD patients. (A) Quantification of GFAP and protein aggregates presence in gray‐matter immunofluorescence images of control and patient samples. A_1_ presents the GFAP integrated density measured in intensity units (i.u). Number of values: N_Controls _= 10, N_Patients _= 12. In A_2_, the scatter plot represents the mean value of Amytracker 680 integrated density in the control and patient groups. Number of values: N_Controls _= 10 control samples, N_Patients _= 12 patient samples. A_3_ shows the average ratio of GFAP to Amytracker 680 integrated density measured in the ROIs studied. Number of values: N_Controls _= 9, N_Patients _= 12. A_4_ indicates the GFAP/Amytracker 680 integrated density ratio for the ROIs of individual subjects by categorizing the patient samples based on BNE stage (III–IV, V, and VI). (B) Representative immunofluorescence images of GFAP (green) and protein aggregates (red, Amytracker 680) in frontal cortex gray‐matter tissue sections. B_1_ shows stained images from control samples. B_2_ shows images from patient samples further stratified by BNE stage (IV, V, and VI). In B, the scale bar is equal to 100 µm. Asterisks indicate statistically significant differences: **, *p*‐value < 0.01. AD, Alzheimer's disease; BNE, BrainNet Europe; GFAP, glial fibrillary acidic protein; ROI, region of interest.

Using the same approach, we assessed astrocytic reactivity in regions of Aβ plaques deposition. As shown in Figure 6A1, GFAP density measured within plaque‐associated ROIs was not different in AD patients compared to controls while the amyloid burden was increased in the AD patients’ group (*p*‐value = 0.009) (Figure 6A2). The latter result agrees with our observation concerning the differences in size and density of the Aβ plaques observed in AD patients compared to those in the controls. Furthermore, the GFAP/Aβ ratio did not differ between the two groups (Figure 6A3). This shows that despite the increased amyloid burden in AD patients, localized astrocytic activation and response were not heightened. Qualitative analysis of immunofluorescence images further supported the above observations. In the control samples (Figure 6B1), we noticed that GFAP‐positive astrocytes aggregated around Aβ plaques, while at the same time the size and density of these plaques were relatively small and low, respectively. In the AD patients, we observed that while GFAP signal localized near Aβ plaques, the staining appeared disorganized and inconsistent across large and dense‐appearing Aβ plaques, suggesting a possible compromised capacity to defend effectively against worsening Aβ pathology (Figure 6B2).

**FIGURE 7:**
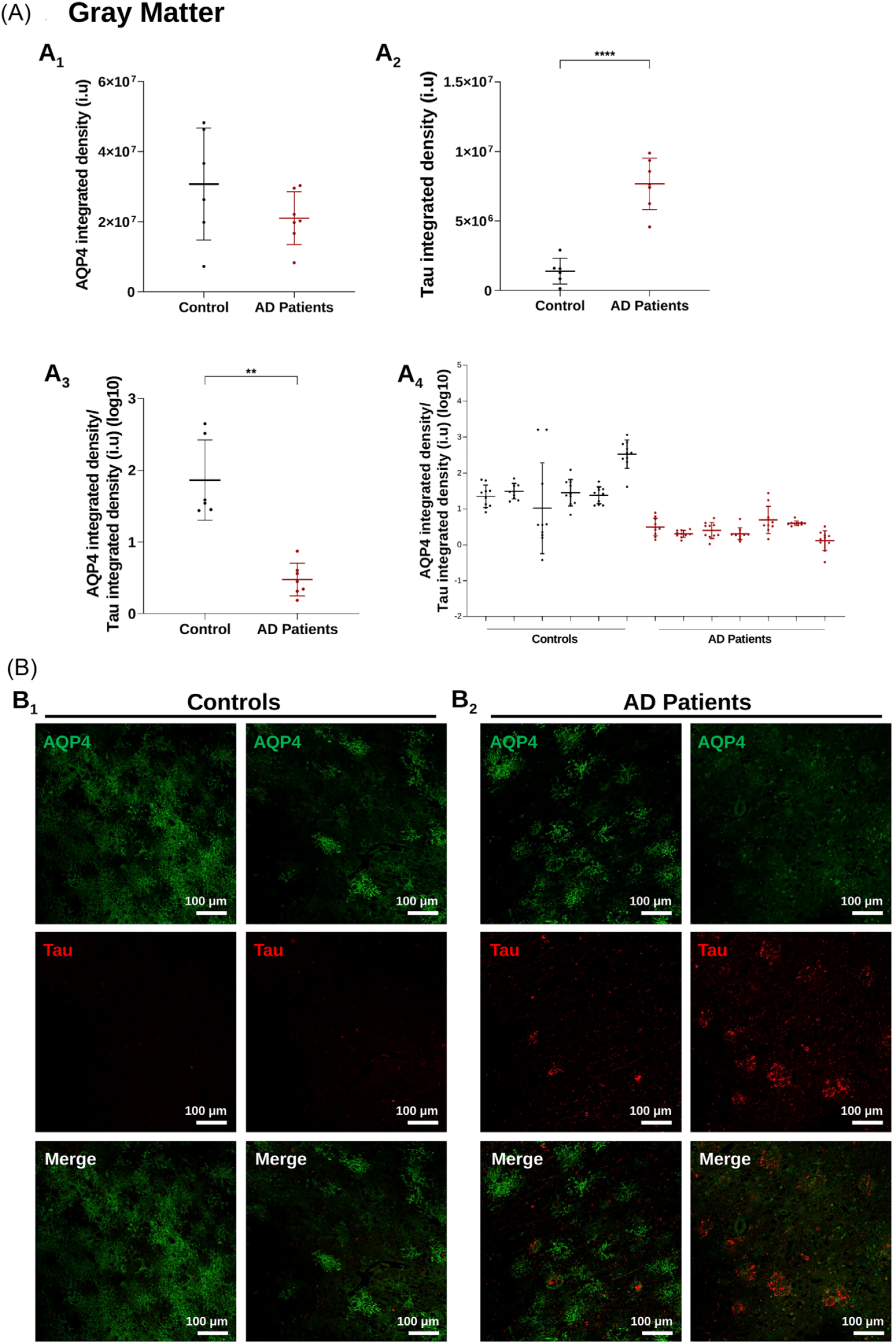
Quantification of AQP4 and tau expression in the frontal cortex gray matter of AD patients. (A) Quantification of AQP4 and tau presence in gray‐matter immunofluorescence images of control and patient samples. A_1_ indicates the AQP4 integrated density measured in intensity units (iu). The scatter plot represents average value calculated from the integrated densities measured in whole 20x magnification images of the respective subject. Number of values: N_Controls _= 6 control samples, N_Patients _= 7 patient samples. A_2_ shows the tau integrated density mean value measured in each sample. Number of values: N_Controls _= 6, N_Patients _= 7. A_3_ shows for each subject the average ratio of AQP4 to tau integrated density measured in the selected images. Number of values: N_Controls _= 6, N_Patients _= 7. A_4_ indicates the AQP4/tau integrated density ratio for the individual subjects. (B) Representative immunofluorescence images of AQP4 (green) and tau (red) in frontal cortex gray‐matter tissue sections. B_1_ shows stained images from control samples. B_2_ shows images from patient samples. In B, the scale bar is equal to 100 µm. Asterisks indicate statistically significant differences: **, *p*‐value < 0.01; ****, *p*‐value < 0.0001. AD, Alzheimer's disease; AQP4, aquaporin‐4.

Finally, to explore the spatial relationship between AQP4 expression and tau pathology, we performed double immunofluorescence staining for AQP4 and tau. Quantification was based on total intensity per channel, without using manually selected ROIs. Image acquisition was restricted to gray‐matter areas, where tau aggregates typically accumulate in AD. AQP4 expression did not differ significantly between groups (*p* = 0.176) (Figure 7A1). Tau signal was robustly increased in AD brain sections (*p*‐value < 0.0001) compared to controls (Figure 7A2), consistent with pathological tau accumulation. When the association between AQP4 and tau was examined, the AQP4/tau ratio was remarkably lower in AD patients (*p*‐value = 0.001) (Figure 7A3), indicating that AQP4 expression is not preserved in regions with high tau burden. Our findings are illustrated by the representative images presented in Figure 7B for controls (B_1_) and patients (B_2_), respectively.

## 4 DISCUSSION

We investigated regional and stage‐dependent alterations of astrocyte‐related proteins AQP4 and GFAP in *post‐mortem* AD frontal cortex. We combined whole‐slide histology and targeted‐immunofluorescence, providing both global and pathology‐centred views of astrocytic changes. Our whole‐slide histology pipeline analyses > 100 images per‐subject capturing the heterogeneity of tissue sections. By separating gray from white matter and stratifying by BNE stage, we captured both regional and stage‐dependent features of astrocyte dysregulation related to Aβ and tau pathology. Our data support a model in which dysregulated AQP4 retention at pathology sites and altered astrocyte reactivity may be relevant to impaired clearance pathways in AD, although glymphatic function and perivascular AQP4 polarization were not directly measured.^16^

Global gray‐matter AQP4 area didn't differ significantly by immunohistochemistry, whereas the AQP4/Aβ and AQP4/tau ratios were significantly reduced, indicating reduced AQP4 relative to local proteinopathy burden. White‐matter AQP4, by contrast, was preserved. This pattern, global preservation versus local reduction around pathology, is consistent with a redistribution rather than a net loss of AQP4 in early‐to‐moderate disease and provides further support for the view that AQP4 changes relevant to glymphatic‐related pathways in AD reflects a pathology‐adjacent reduction.

Our findings are comparable to key human *post‐mortem* studies. Zeppenfeld ^8^ reported increased global gray‐matter AQP4 in AD relative to young and aged controls, together with increased AQP4 immunoreactivity surrounding amyloid plaques, in the setting of loss of perivascular polarization. In contrast, our global gray‐matter AQP4 showed a non‐significant downward trend, and although AQP4 rose at BNE stage VI, this increase reached significance only relative to earlier AD stages and didn't exceed control levels. We show concordance with Simon,^9^ who reported no significant difference in total AQP4 area coverage. Critically, our pathology‐anchored measure, the plaque‐ and tau‐adjacent AQP4/aggregate ratio, is the metric most comparable to the peri‐plaque AQP4 quantified by Zeppenfeld.^8^ We observed reduced, not increased, AQP4 around pathology (lower AQP4/Aβ and AQP4/tau ratios).

These divergences may be methodological rather than biological. Zeppenfeld ^8^ quantified immunofluorescence intensity and Simon ^9^ a vessel‐anchored perivascular polarization index, whereas our whole‐slide analysis reports DAB OD and a pathology‐anchored AQP4/aggregate ratio, measurements with different dynamic ranges. Notably, the apparent difference with Zeppenfeld ^8^ narrows when considering their Western blot data, which found no significant difference in total AQP4, limiting the divergence to their intensity measure. The peri‐plaque comparison is likewise indirect. The peri‐plaque AQP4 of Zeppenfeld ^8^ was a qualitative, within‐AD observation, whereas ours is a between‐group integrated‐density comparison across amyloid‐positive individuals. Cohort composition, and our sampling of over 100 fields across full cortical depth, further limit direct quantitative comparison.

Our observation of a selective, pathology‐adjacent reduction of gray‐matter AQP4 agrees with reports of selective vulnerability ^17^, ^18^ of gray‐matter astrocytes to remodeling. In rodents, AQP4 deletion compromises CSF–ISF exchange and accelerates Aβ deposition,^5^, ^19^ while mislocalization alone suffices. In α‐syntrophin‐deficient mice, AQP4 is displaced from perivascular endfeet despite preserved expression, and this loss of perivascular localization is associated with reduced CSF influx. In amyloidosis background, altered AQP4 localization is associated with increased Aβ deposition.^6^, ^9^ *Post‐mortem* studies similarly show that reduced perivascular AQP4 localization correlates with increased Aβ and tau pathology.^8^, ^9^

Stratification of AQP4 expression by BNE stage revealed increased AQP4 levels at BNE stage VI in both gray and white matter, despite no evidence of increased amyloid in the gray matter. This likely reflects a compensatory or inflammatory gliotic response, in which AQP4 expression increases in later‐stage disease while its functional localization likely remains disrupted. Crucially, in plaque‐centred ROIs the AQP4/Aβ ratio remained depressed across all stages, indicating that the late‐stage rise reflects surrounding amyloid pathology. Our immunofluorescence refined this interpretation by showing that local AQP4/Aβ ratios were significantly reduced in AD, irrespective of BNE, suggesting a sustained pathology‐adjacent reduction of AQP4. This late‐stage upregulation is compatible with observations in the tg‐ArcSwe mouse model of amyloidosis.^20^

Another distinction concerns disease trajectory. Whereas prior studies described AQP4 change progressing with Braak/BNE stage, in our cohort the plaque‐adjacent AQP4/Aβ ratio is already maximally depressed by BNE III–IV and changes little thereafter, suggesting that the pathology‐adjacent AQP4 reduction is an early, saturating event rather than one tracking end‐stage tau burden.

We further demonstrated a reduction in AQP4 expression relative to tau, indicating that AQP4 expression is inversely correlated to neurofibrillary pathology. Our data indicate that tau‐driven astrocytic structural remodeling is associated with reduced AQP4‐retention in tau‐rich regions. Whether this reduction extends to the perivascular compartment and thereby exacerbates interstitial stagnation and tau propagation, merits further investigation. This interpretation is consistent with recent in‐vitro findings showing that phosphorylated tau accumulation can directly impair glymphatic clearance by inducing astrocytic morphological changes, leading to vasoconstriction and reduced perivascular flow.^21^ Pathology‐adjacent AQP4 reduction in AD may function both as contributor and consequence of proteinopathy, establishing a self‐reinforcing cycle of impaired clearance and aggregate accumulation.^22^

Astrocyte reactivity, marked by increased GFAP expression, is a hallmark of AD neuroinflammation ^23^, ^24^ with plaque‐associated astrogliosis documented in humans and rodents.^25^ Our immunohistochemistry revealed divergent GFAP responses across cortical compartments. In white matter, both univariate and regression analyses identified the AD group as a significant predictor of reduced GFAP concentration. In gray‐matter, GFAP Mean DAB Intensity was significantly decreased in AD, whereas GFAP area–fraction trended upward and was strongly sex‐influenced. This area–intensity dissociation is consistent with redistribution of GFAP into more numerous but less filament‐dense reactive astrocytes, matching the fragmented peri‐plaque labeling seen by immunofluorescence.

Under physiological conditions, GFAP expression is higher in fibrous (white‐matter) astrocytes than protoplasmic (gray‐matter) astrocytes.^26^ Protoplasmic astrocytes contribute to synaptic regulation and metabolic support, whereas fibrous astrocytes specialize in maintaining ionic homeostasis along myelinated axons interacting with oligodendrocytes.^27^ Thus, the observed reduction in GFAP may reflect white‐matter myelin or axonal pathology aligning with evidence indicating that white‐matter astrocytes may contribute to the underappreciated axonal and myelin AD degeneration.^28^ Astrocyte–oligodendrocyte uncoupling, or altered gap‐junction communication may underlie this phenotype.^29^ Consistently, serum GFAP is an emerging biomarker of AD reactive astrogliosis, reflecting multiple convergent mechanisms of astrocytic remodeling.^30^

By our immunofluorescence, the GFAP/Aβ intensity ratio in plaque‐associated gray‐matter did not differ between AD and controls. This indicates that local astrocytic activation does not scale with amyloid burden, suggesting a maladaptive or exhausted reactive state. This aligns with transcriptomics showing disease‐associated astrocyte subtypes in AD, with upregulated inflammatory and reactive markers such as GFAP, downregulation of homeostatic genes, and impaired Aβ clearance capacity.^31^

Consistent with these signatures, our imaging showed GFAP‐positive astrocytes aggregated around Aβ‐plaques in controls, when these plaques were relatively small, forming organized boundaries. In contrast, in AD, GFAP labeling appeared disorganized around large, dense amyloid deposits, suggesting a compromised capacity to respond to Aβ pathology. This may reflect a maladaptive reactive state,^32^ possibly corresponding to the A1 neurotoxic astrocyte phenotype, which is prevalent in human AD brain tissue,^33^ Together, these findings suggest a diminished capacity for coordinated astrocytic response and exhaustion or senescence of astrocytes in regions of advanced pathology.^34^, ^35^

A major strength of our study is the explicit anatomical distinction between gray and white‐matter astrocytes, an often‐overlooked factor in human neuropathology. A novel observation was the enrichment of GFAP‐positive astrocytes along the gray–white matter border in AD, coinciding with the accumulation of large and mature plaques at this interface. AQP4 expression terminated at this boundary. Whether this glial border represents a passive consequence of fluid stagnation at a glymphatic interface or an active barrier warrants investigation and highlights the gray‐white interface as an under‐appreciated point of astrocyte‐mediated AD pathology.

Clinically, CSF and plasma GFAP and AQP4 are emerging biomarkers of AD progression. Our findings suggest they are most informative when interpreted by compartment and disease stage, and—for AQP4—when considering localization rather than absolute levels. These results also underscore the therapeutic potential of restoring AQP4 polarization, with neuro‐modulatory stimulation ^36^ or pharmacologic modulation^37^ and reprogramming astrocyte reactivity toward a neuroprotective A2 phenotype.^38^, ^39^, ^40^

Limitations of our study include modest sample sizes within each BNE stage and the use of single‐marker immunohistochemistry, which precludes multiplex discrimination of astrocyte subtypes or direct assessment of perivascular AQP4 localization. Also, our pipeline cannot distinguish perivascular from parenchymal AQP4; therefore, our pathology‐adjacent AQP4 ratios should not be interpreted as the AQP4 polarization indices that prior studies ^8^, ^9^ identified as key pathological features of AQP4 in AD. The dissociation we observe between preserved or elevated total AQP4 and reduced plaque‐ and tau‐adjacent AQP4 is consistent with, but does not directly demonstrate, a loss of perivascular polarity.

In conclusion, AD human frontal cortex demonstrates an AQP4 preservation pattern in gray matter, with late‐stage rises in both gray and white matter, set against a significant reduction of AQP4/Aβ and AQP4/tau ratios, consistent with reduced AQP4 retention relative to local proteinopathy burden. GFAP shows a compartment‐specific phenotype: intensity is significantly reduced in both gray and white matter, while area fraction trends upward in gray matter with qualitatively disorganized peri‐plaque morphology. These findings identify a dissociation between global AQP4 abundance and its retention at proteinopathy sites as a feature of AD pathophysiology.

## AUTHOR CONTRIBUTIONS

H.A.: Conception and design of the study; interpretation of data; acquisition and analysis of data; drafting of the manuscript and figures; revision of manuscript. X.L.: Design of the study; interpretation of data; acquisition and analysis of data; drafting of the manuscript and figures. E.R.D.N: Design of the study; Interpretation of data; revision of manuscript. K.K.: Acquisition of data. L.C.: Interpretation of data. M.T.P.: Interpretation of data; revision of manuscript. I.P.T.: Resources; Revision of manuscript H.W.: Design of the study; interpretation of data; revision of manuscript. M.P: Chief investigator of the research program; conception and design of the study; secured funding for the study; interpretation of data; revision of manuscript.

## CONFLICT OF INTEREST STATEMENT

All authors do not report any conflicts of interest. Author disclosures are available in the Supporting Information.

## CONSENT STATEMENT

Consent was not necessary.

## Supporting information



Supporting information: alz71745‐sup‐0001‐SuppMat.docx

Supporting information: alz71745‐sup‐0002‐Disclosureforms.pdf

Supporting information: alz71745‐sup‐0003‐SuppMat.pdf

## Data Availability

The custom Python scripts developed and utilized for digital image analysis have been uploaded to a public GitHub repository and are available at: https://github.com/xanthippilouka/Human_Brain_Image_Analysis.git. The raw datasets analyzed during the current study are available from the corresponding author on reasonable request.
