# Supplementary material for: Regional astrocyte dysregulation and altered glymphatic‐related markers in Alzheimer's disease frontal cortex
Source: Alzheimers Dement. 2026 Aug 14;22(8):e71745. doi: 10.1002/alz.71745 (PMC13474155; doi:10.1002/alz.71745)
Supplement: Supplementary file 2 [file ALZ-22-e71745-s002.pdf]

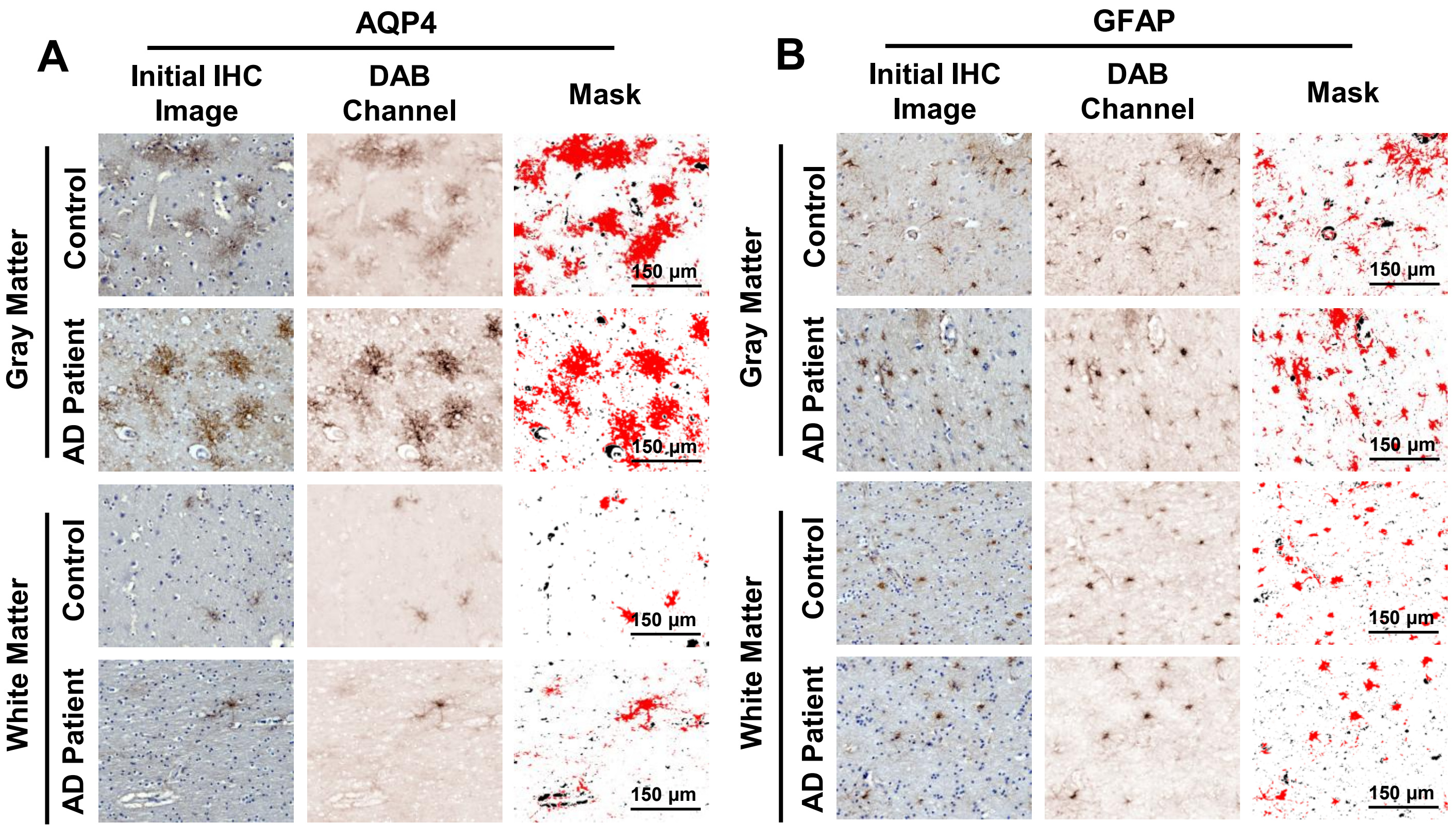

Suppl. Figure 1. Image Processing Pipeline for Astroglial Markers Quantification.

The automated analysis workflow is illustrated for (**A**) AQP4 and (**B**) GFAP across gray and white matter compartments in both Control and AD Patient subjects. In the first column, the initial IHC image is presented; DAB-stained (brown) sections counterstained with hematoxylin (blue). In the second column, the DAB channel is presented; isolated chromogen signal following color deconvolution. In the third column, the generated Mask is presented; binary representation of the identified DAB-positive pixels (red), and DAB-negative pixels (white). The black-colored pixels represent the non-tissue pixels. The scale bar is equal to 150 µm.

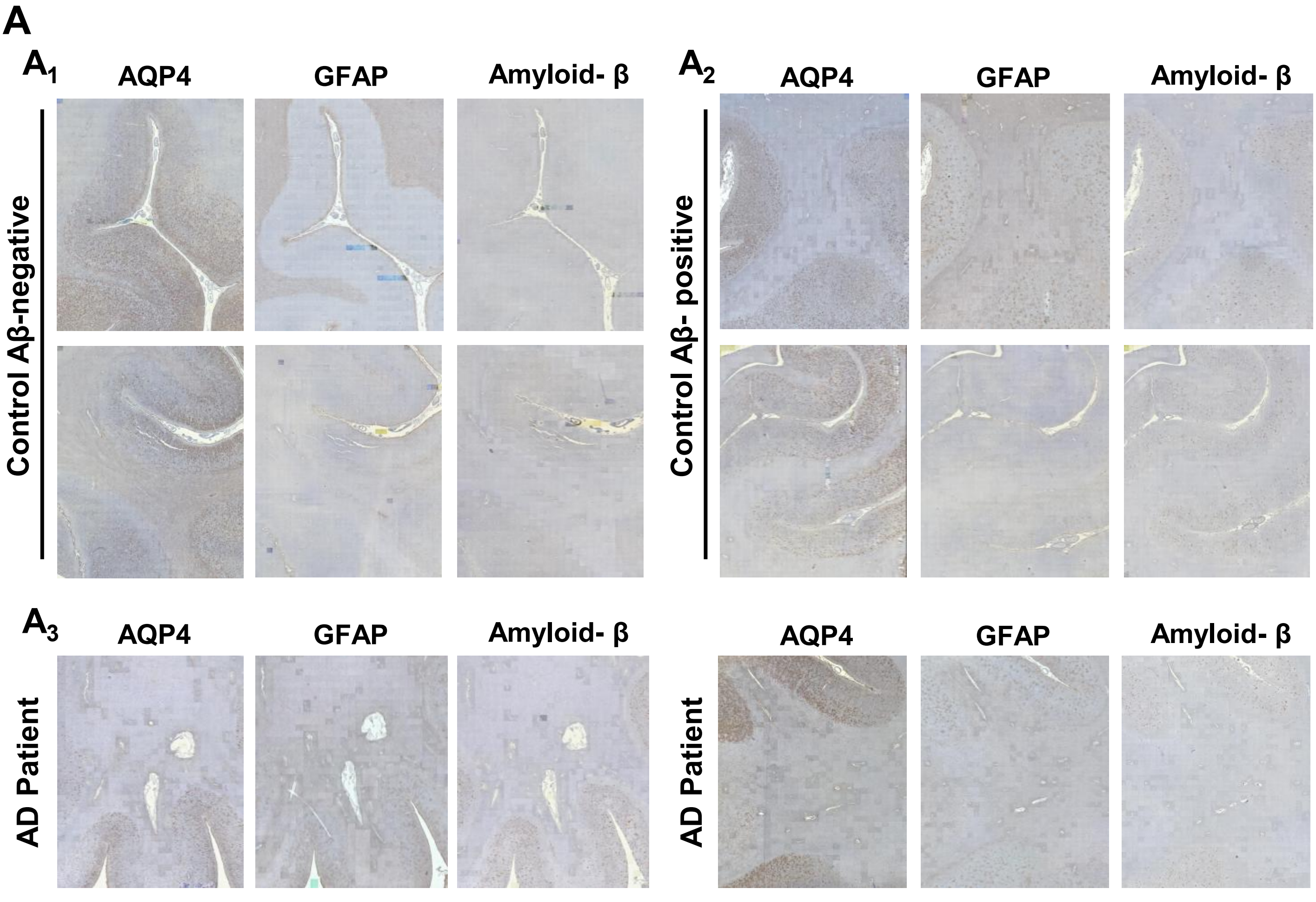

Suppl. Figure 2. Representative Whole-Slide Images for AQP4, GFAP, Amyloid-β Staining.

A. Representative whole-slide images of immunohistochemical staining for AQP4, GFAP, and Amyloid-β in human brain frontal cortex tissue sections from Control Aβ-negative samples (**A_1**), Control Aβ-positive samples (**A_2**), and AD Patient samples (**A_3**).

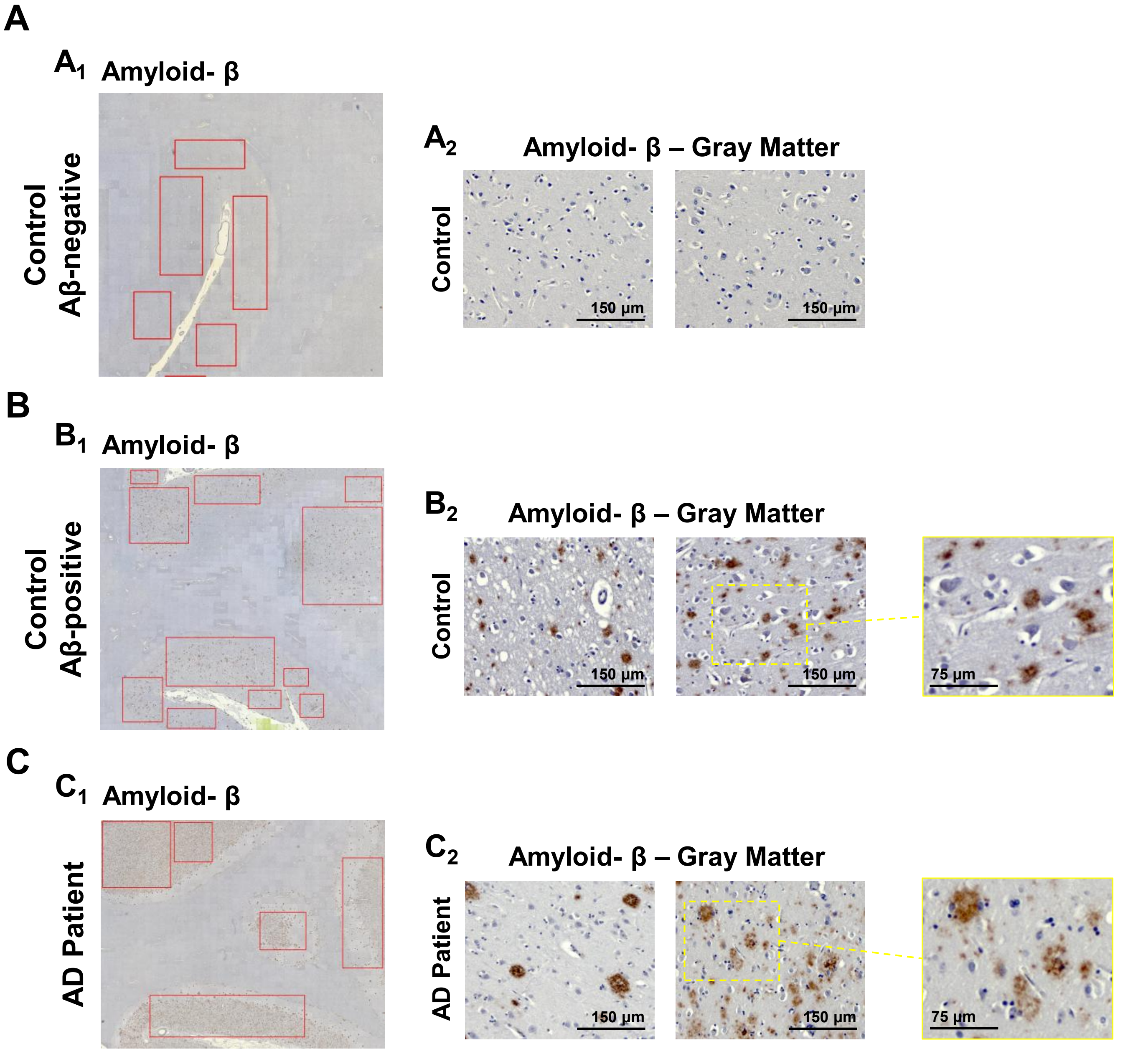

Suppl. Figure 3. Amyloid-β Expression in the Frontal Cortex.

Representative whole slide images of immunohistochemical staining for Amyloid-β in human brain frontal cortex tissue sections from a Control Aβ-negative sample (**A$_1$**), a Control Aβ-positive sample with observed Aβ plaques (**B$_1$**), and an AD Patient sample (**C$_1$**). Representative higher magnification (20x objective) immunohistochemical images of Aβ expression in the gray matter of a Control Aβ-negative sample (**A$_2$**), a Control Aβ-positive sample (**B$_2$**), and an AD Patient sample (**C$_2$**). Brown staining indicates Aβ positive regions. Yellow dashed boxes indicate ROIs shown as high-magnification insets below. The scale bar is equal to 150 µm (main images) and 75 µm (insets).

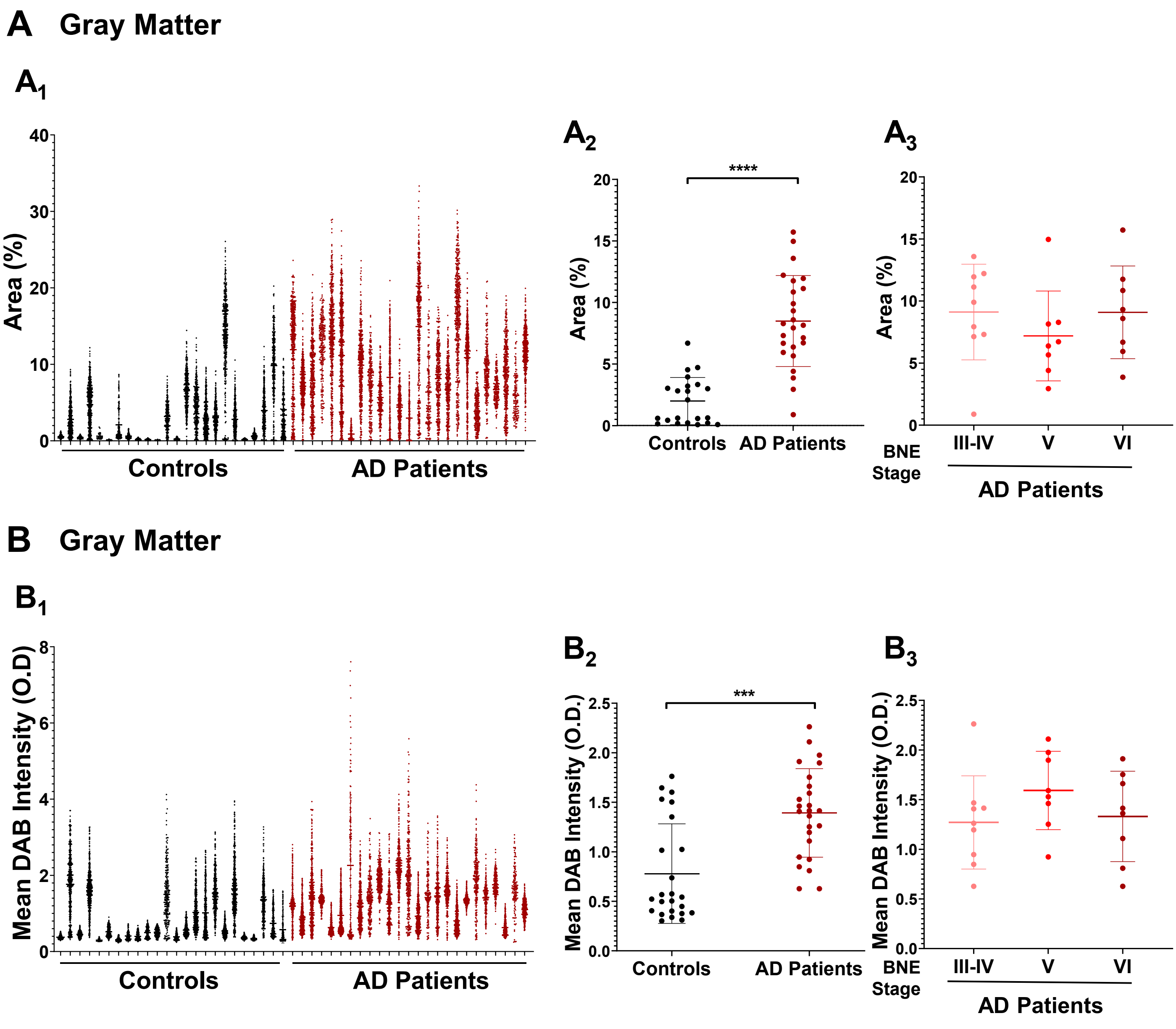

Suppl. Figure 4. Quantitative analysis of Amyloid-β Expression in the Frontal Cortex Gray Matter.

(**A**$_1$, **B**$_1$) Scatter plots show the distribution of the Aβ Area (%) and Mean Aβ Intensity, respectively, calculated for each immunohistochemical image (20x magnification) from each subject; control or AD patient respectively. (**A**$_2$, **B**$_2$) Scatter plots present the average value of the Aβ Area (%) and Mean Aβ Intensity for each subject of the control and patient group. Number of values: $N_{Controls} = 22$, $N_{Patients} = 25$ for the Area (%) and $N_{Controls} = 24$, $N_{Patients} = 25$ for Mean DAB Intensity. (**A**$_3$, **B**$_3$) Scatter plots indicate the average value of the above metrics in each patient grouped by BNE stage (III-IV, V, and VI). Number of values: $N_{III\text{-}IV} = 9$, $N_V = 8$, and $N_{VI} = 8$ for both metrics. Asterisks indicate statistically significant differences: ***, P-value < 0.001; ****, P-value < 0.0001.

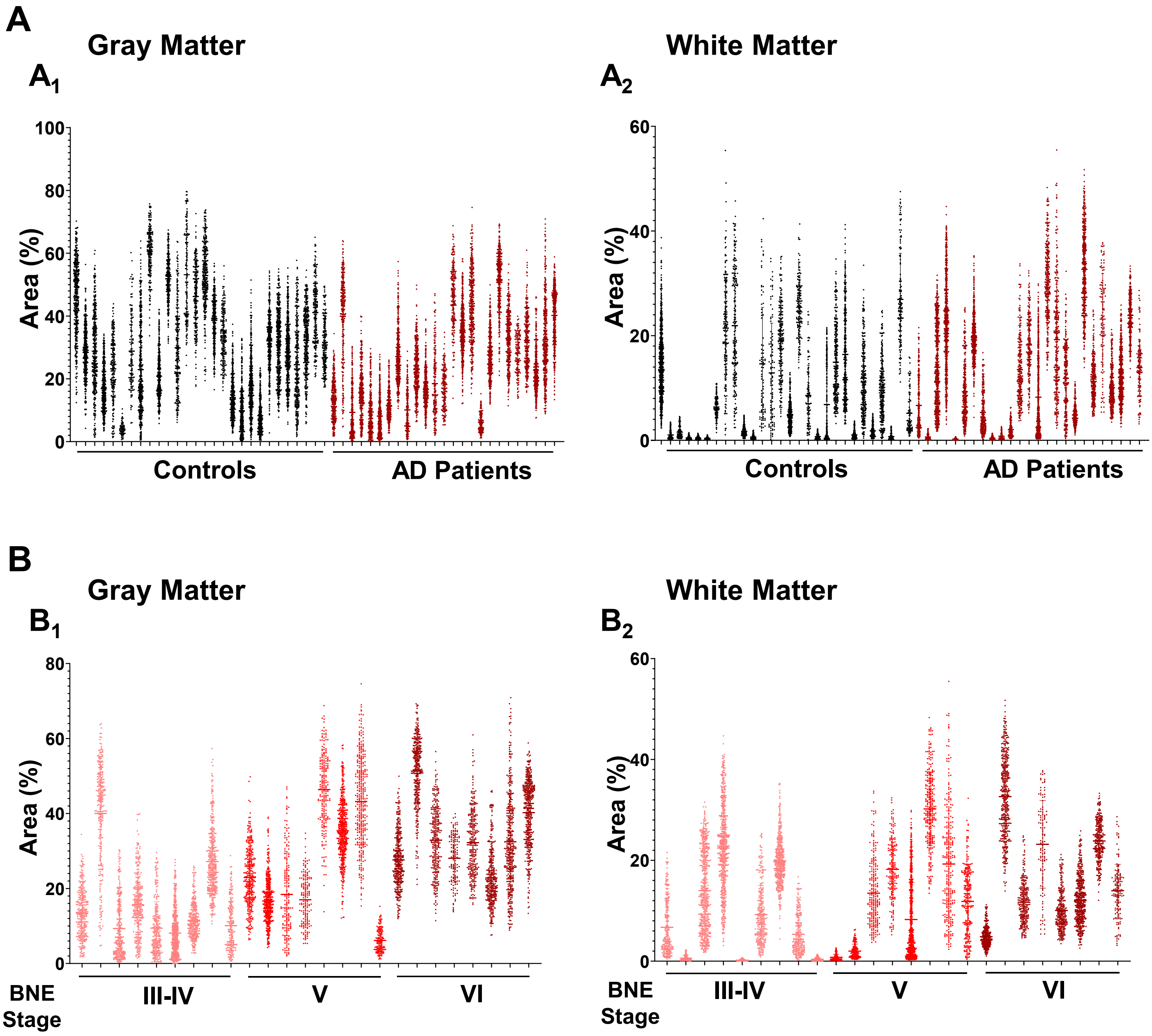

Suppl. Figure 5. AQP4 Coverage in the Frontal Cortex.

A. Analysis of gray and white matter 20x magnification images, respectively. Scatter plots show the distribution of the AQP4 Area (%) calculated for each immunohistochemical image in gray (**A_1**) and white (**A_2**) matter, respectively, for each subject from the control and AD patient group. Each point in the graph represents the respective value obtained from each subject's selected immunohistochemical images (20x magnification). **B.** Scatter plots display the AQP4 Area (%) in gray (**B_1**) and white (**B_2**) matter counted in selected images from the studied AD patients stratified by BNE stage (III-IV, V, and VI).

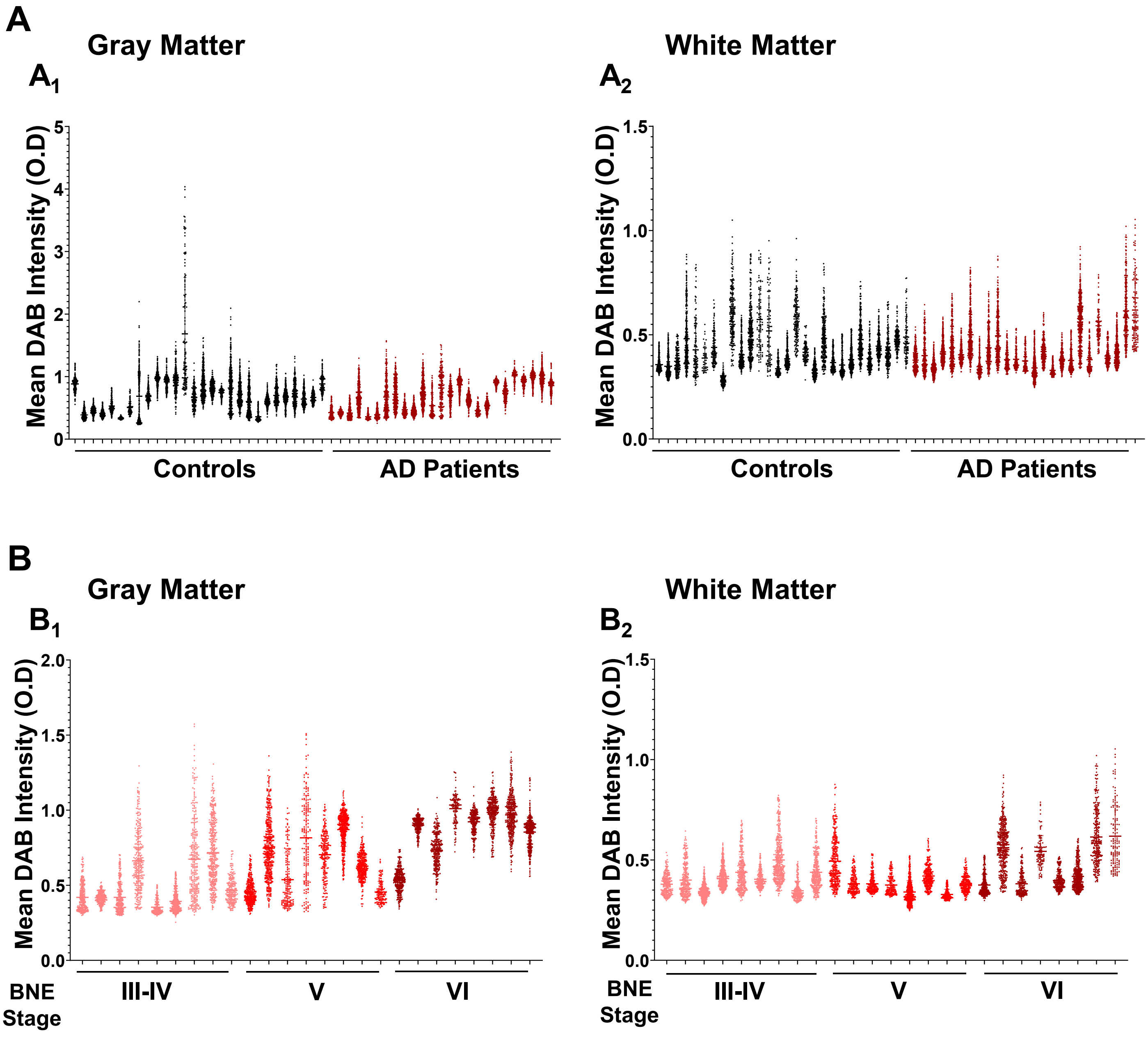

Suppl. Figure 6. AQP4 Concentration per pixel in the Frontal Cortex.

A. Analysis of gray and white matter images, respectively. Scatter plots show the distribution of the Mean AQP4 Intensity calculated for each immunohistochemical image in gray (**A_1**) and white (**A_2**) matter, respectively, for each subject from the control and AD patient group. Each point in the graph represents the respective value obtained from each subject's selected immunohistochemical images (20x magnification). **B.** Scatter plots display the Mean AQP4 Intensity in gray (**B_1**) and white (**B_2**) matter, counted in selected images from the studied AD patients stratified by BNE stage (III-IV, V, and VI).

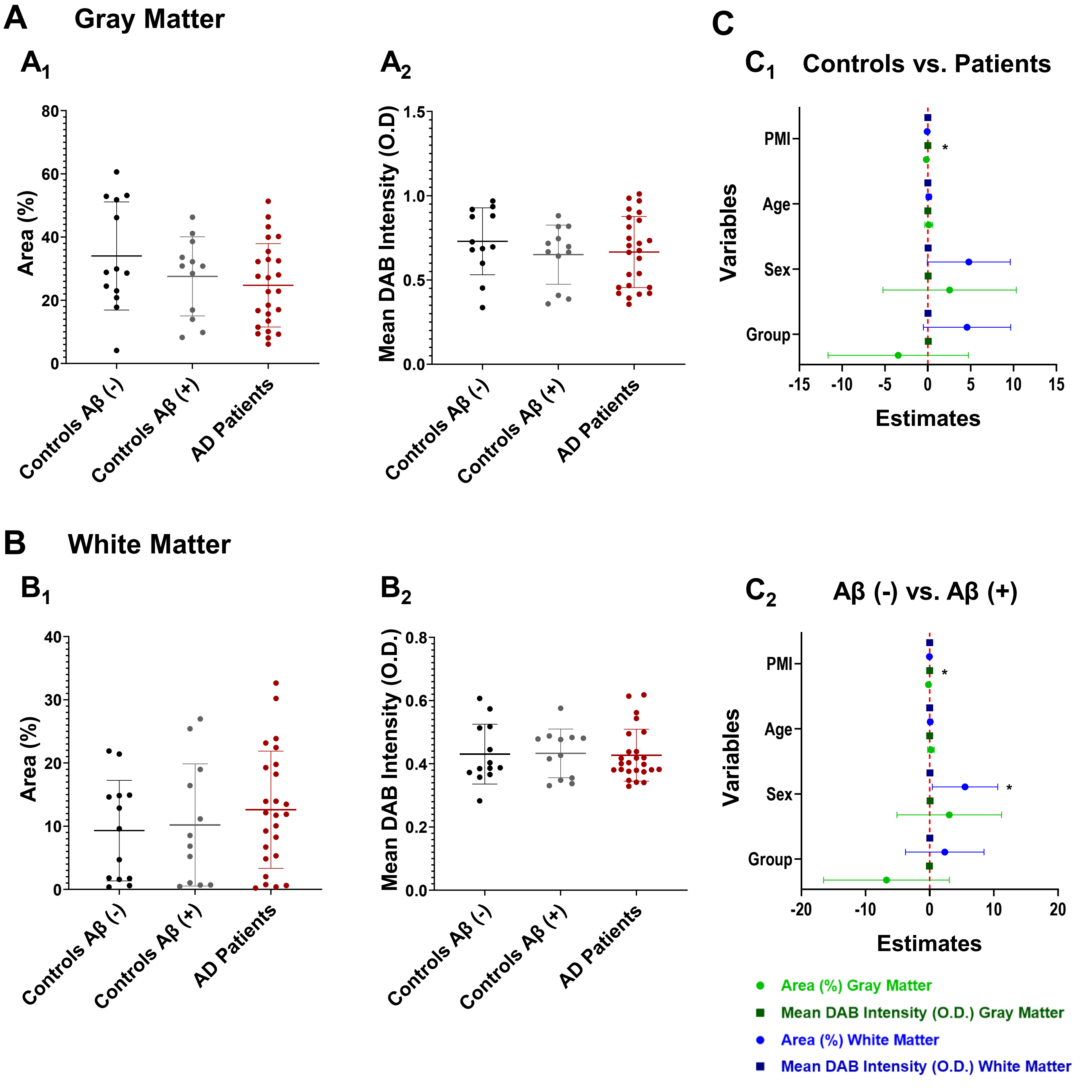

Suppl. Figure 7. Impact of Amyloid status and clinical features on AQP4 expression.

A. Gray matter analysis. Quantification of AQP4 Area (%) (**A_1**) and Mean DAB Intensity (**A_2**) in gray matter across stratified groups: Controls Aβ-negative (black), Controls Aβ-positive (grey), and AD Patients (red). $N_{Aβ(-)} = 13$, $N_{Aβ(+)} = 12$, $N_{Patients} = 25$ for Area (%) and $N_{Aβ(-)} = 12$, $N_{Aβ(+)} = 12$, $N_{Patients} = 25$ for Mean DAB Intensity. **B.** White matter analysis. Quantification of AQP4 Area (%) (**B_1**) and Mean DAB Intensity (**B_2**) in white matter across stratified groups: Controls Aβ-negative, Controls Aβ-positive, and AD Patients. $N_{Aβ(-)} = 13$, $N_{Aβ(+)} = 12$, $N_{Patients} = 25$ for both metrics. **C.** Multiple linear regression models. (**C_1**) Forest plot shows coefficient estimates for the global comparison model (Controls vs AD Patients). (**C_2**) Forest plot for the amyloid-stratified model (Aβ-negative vs Aβ-positive). *, P-value < 0.05.

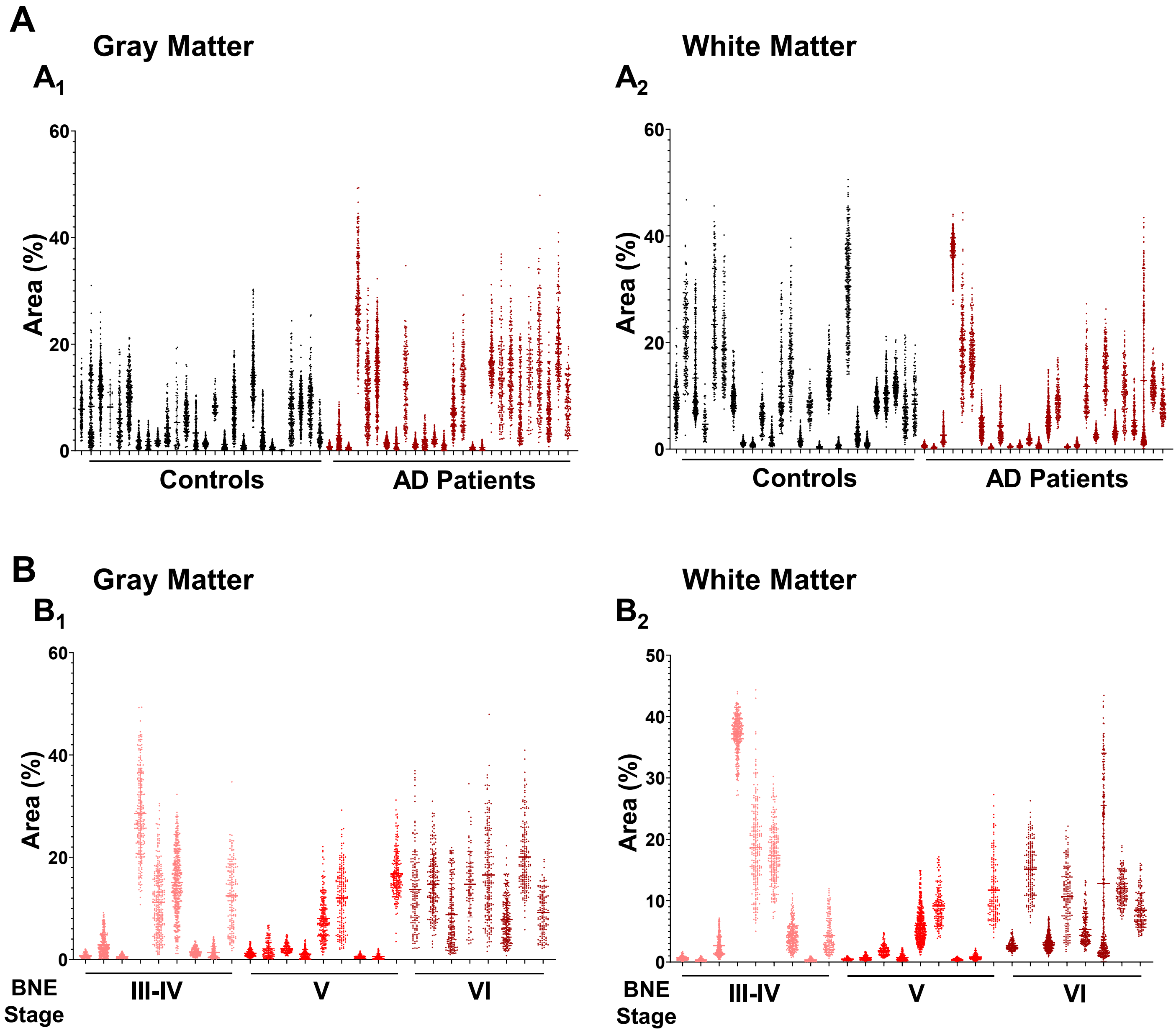

Suppl. Figure 8. GFAP Coverage in the Frontal Cortex.

A. Analysis of gray and white matter 20x magnification images. Scatter plots show the distribution of the GFAP Area (%) calculated for each immunohistochemical image in gray (**A_1**) and white (**A_2**) matter, respectively, for each subject from the control and AD patient group. Each point in the graph represents the respective value obtained from each subject's selected immunohistochemical images (20x magnification). **B.** Scatter plots display the GFAP Area (%) in gray (**B_1**) and white (**B_2**) matter counted in selected images from the studied AD patients stratified by BNE stage (III-IV, V, and VI).

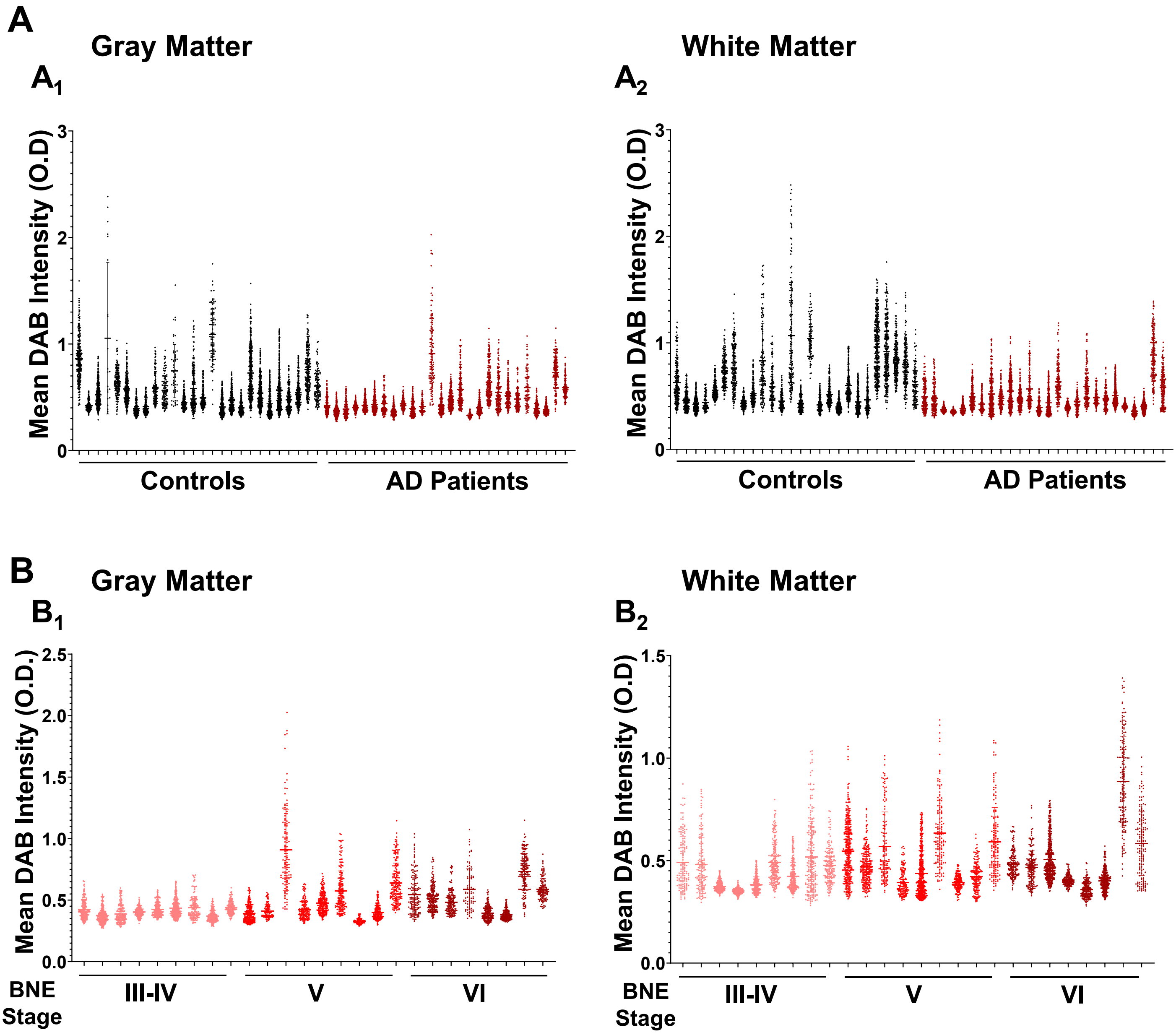

Suppl. Figure 9. GFAP Concentration per pixel in the Frontal Cortex.

A. Analysis of gray and white matter images. Scatter plots show the distribution of the Mean GFAP Intensity calculated for each immunohistochemical image in gray (**A_1**) and white (**A_2**) matter, respectively, for each subject from the control and AD patient group. Each point in the graph represents the respective value obtained from each subject's selected immunohistochemical images (20x magnification). **B.** Scatter plots display the Mean GFAP Intensity in gray (**B_1**) and white (**B_2**) matter, counted in selected images from the studied AD patients stratified by BNE stage (III-IV, V, and VI).

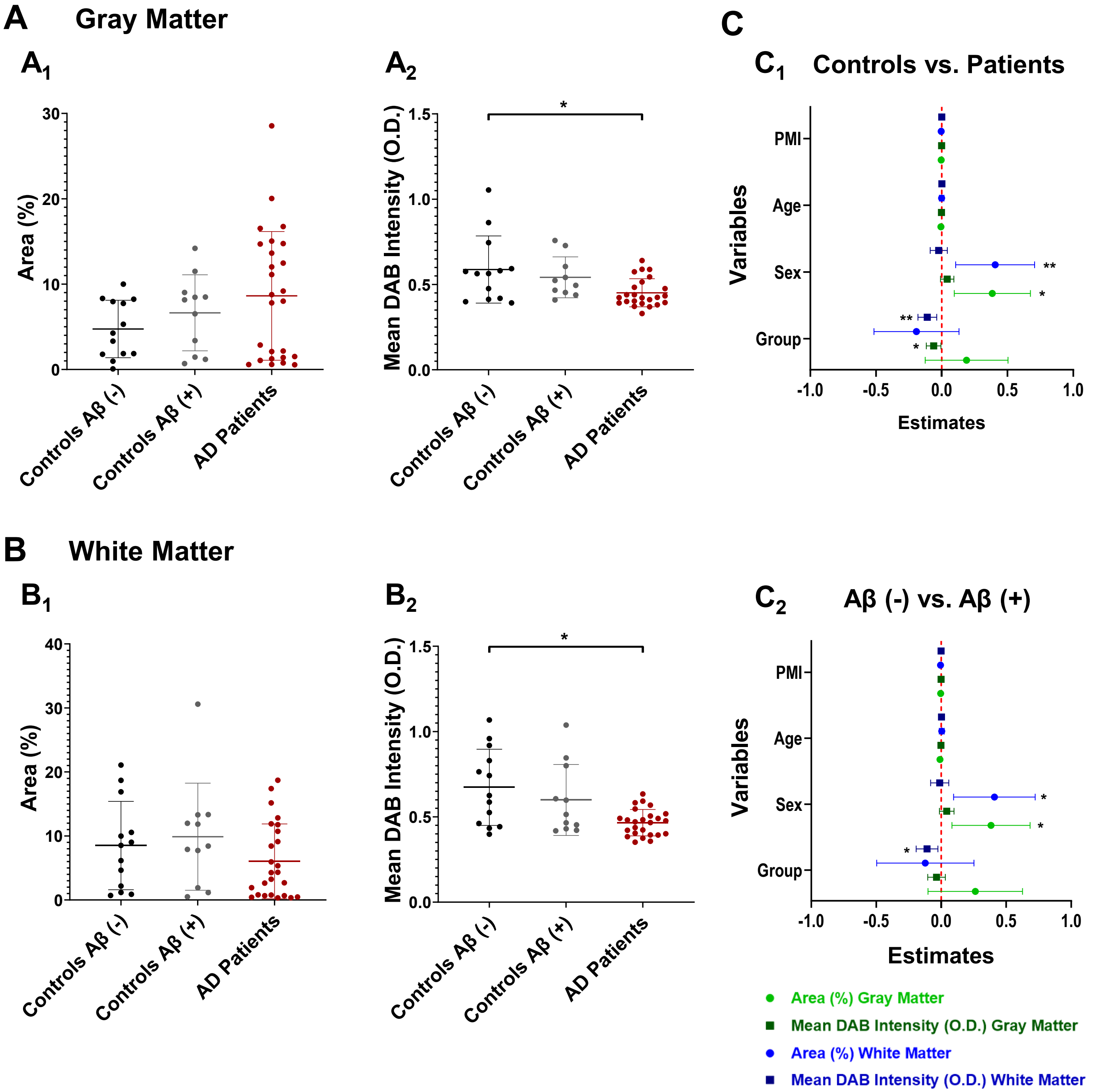

Suppl. Figure 10. Impact of Amyloid status and clinical features on GFAP expression.

A. Gray matter analysis. Quantification of GFAP Area (%) (**A$_1$**) and Mean DAB Intensity (**A$_2$**) in gray matter across stratified groups: Controls Aβ-negative (black), Controls Aβ-positive (grey), and AD Patients (red). $N_{A\beta(-)} = 13$, $N_{A\beta(+)} = 11$, $N_{Patients} = 26$ for Area (%) and $N_{A\beta(-)} = 13$, $N_{A\beta(+)} = 10$, $N_{Patients} = 24$ for Mean DAB Intensity. **B.** White matter analysis. Quantification of GFAP Area (%) (**B$_1$**) and Mean DAB Intensity (**B$_2$**) in white matter across stratified groups: Controls Aβ-negative, Controls Aβ-positive, and AD Patients. $N_{A\beta(-)} = 13$, $N_{A\beta(+)} = 11$, $N_{Patients} = 25$ for both metrics. **C.** Multiple linear regression models. (**C$_1$**) Forest plot shows coefficient estimates for the global comparison model (Controls vs AD Patients). (**C$_2$**) Forest plot for the amyloid-stratified model (Aβ-negative vs Aβ-positive). *, P-value < 0.05; **, P-value < 0.001.

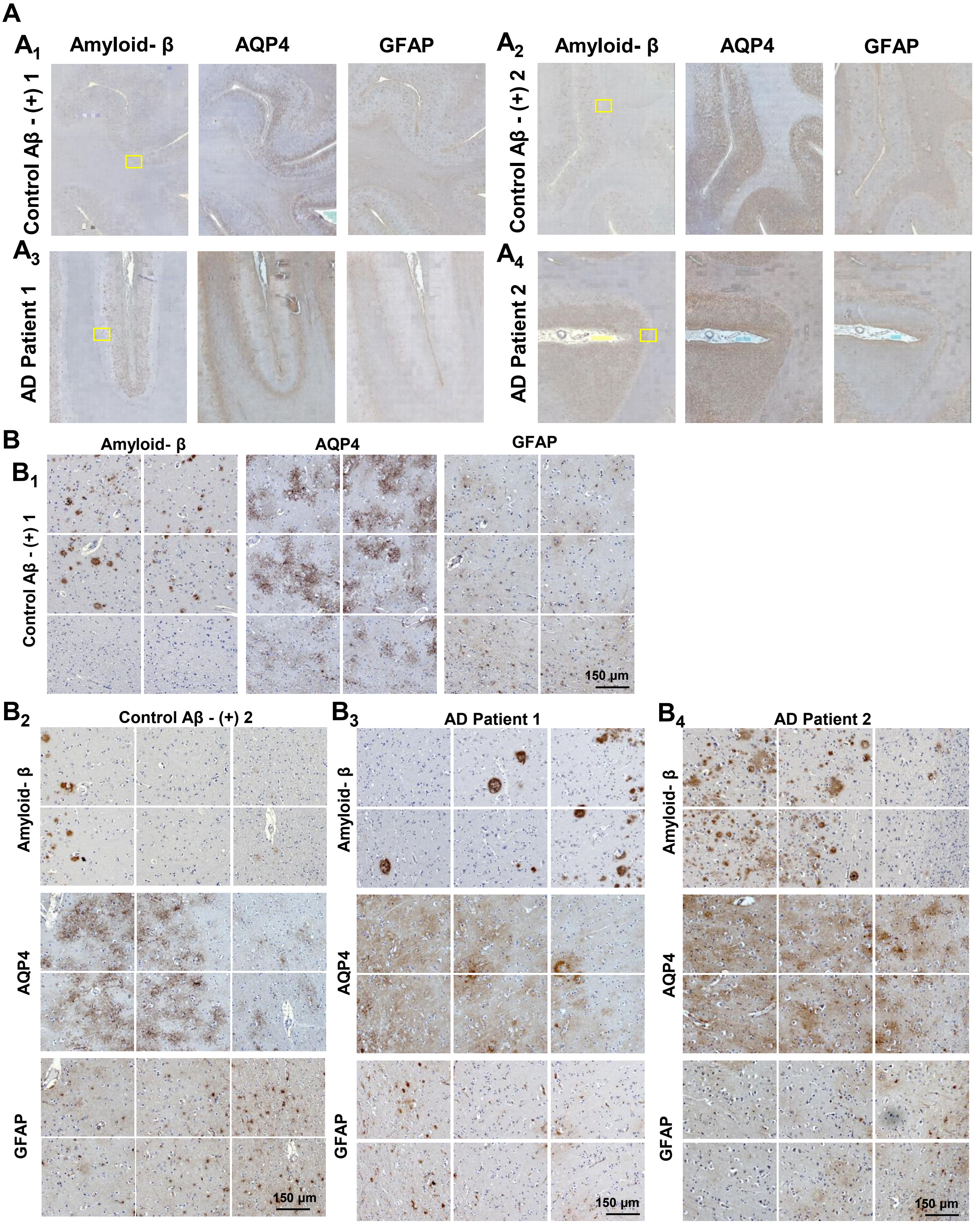

Suppl. Figure 11. Distribution of Amyloid-β, AQP4, and GFAP across the Gray-White matter Interface.

A. Representative whole-slide IHC images illustrate the spatial organization of Aβ pathology and glial markers (AQP4, GFAP) in Control Aβ-(+) ($\mathbf{A}_{1,2}$) and AD patients ($\mathbf{A}_{3,4}$) groups. Each row displays a triplet of images obtained from the same subject to illustrate the spatial inter-relationship between pathology and glial architecture. Yellow boxes indicate the regions shown in higher magnification in (**B**).

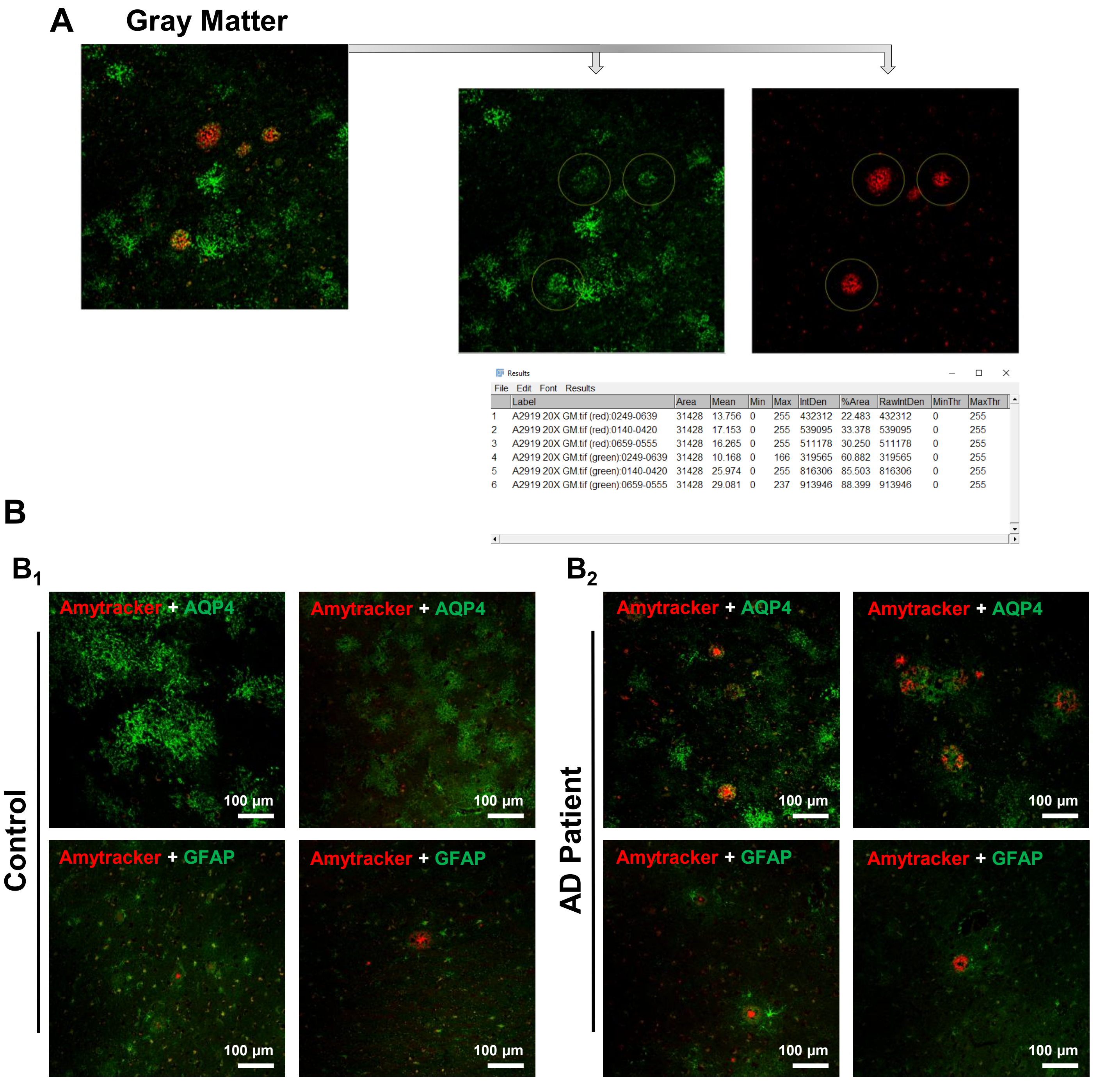

Suppl. Figure 12. Immunofluorescence Analysis of Proteins under interest around Regions of Protein Accumulation in the Frontal Cortex Gray Matter.

A. Methodology of selecting regions of interest (ROIs) for AQP4 or GFAP expression analysis around protein aggregates. Using Fiji image processing software, oval ROIs (Width: 200 pixels, Height: 200 pixels) were designed around observed protein aggregates stained with Amytracker 680, and the respective integrated density for the green (AQP4/GFAP staining) and red (Amytracker staining) channels for each ROI was measured. **B.** Representative immunofluorescence images (20x magnification) of AQP4 (green) and Amytracker 680 (red) co-staining of controls' (**B$_1$**) and patients' (**B$_2$**) frontal cortex gray matter tissue sections. The scale bar is equal to 100 µm.
