# Supplementary material for: Regional astrocyte dysregulation and altered glymphatic‐related markers in Alzheimer's disease frontal cortex
Source: Alzheimers Dement. 2026 Aug 14;22(8):e71745. doi: 10.1002/alz.71745 (PMC13474155; doi:10.1002/alz.71745)
Supplement: Supplementary file 3 [file ALZ-22-e71745-s003.docx]

| **AD Patients** | | | |  | **Controls** | | | |
| --- | --- | --- | --- | --- | --- | --- | --- | --- |
| **ID** | **AQP4** | **GFAP** | **Abeta42** |  | **ID** | **AQP4** | **GFAP** | **Abeta42** |
| **AD1** | ✔ | ✔ | ✔ |  | **C1** | ✔ | ✔ | ✔ |
| **AD2** | ✔ | ✔ | ✔ |  | **C2** | ✔ | ✔ | ✔ |
| **AD3** | ✔ | ✔ | ✔ |  | **C3** | ✔ | ✔ |  |
| **AD4** | ✔ | ✔ | ✔ |  | **C4** | ✔ |  |  |
| **AD5** | ✔ | ✔ | ✔ |  | **C5** | ✔ | ✔ | ✔ |
| **AD6** | ✔ | ✔ | ✔ |  | **C6** | ✔ | ✔ | ✔ |
| **AD7** | ✔ | ✔ | ✔ |  | **C7** | ✔ | ✔ | ✔ |
| **AD8** | ✔ | ✔ | ✔ |  | **C8** | ✔ | ✔ | ✔ |
| **AD9** | ✔ | ✔ | ✔ |  | **C9** | ✔ | ✔ | ✔ |
| **AD10** | ✔ | ✔ | ✔ |  | **C10** | ✔ |  | ✔ |
| **AD11** | ✔ | ✔ |  |  | **C11** | ✔ | ✔ | ✔ |
| **AD12** | ✔ | ✔ | ✔ |  | **C12** | ✔ | ✔ | ✔ |
| **AD13** | ✔ | ✔ | ✔ |  | **C13** | ✔ | ✔ | ✔ |
| **AD14** | ✔ | ✔ | ✔ |  | **C14** | ✔ | ✔ | ✔ |
| **AD15** | ✔ | ✔ | ✔ |  | **C15** | ✔ | ✔ | ✔ |
| **AD16** |  | ✔ | ✔ |  | **C16** | ✔ | ✔ | ✔ |
| **AD17** | ✔ | ✔ | ✔ |  | **C17** | ✔ | ✔ | ✔ |
| **AD18** | ✔ | ✔ | ✔ |  | **C18** | ✔ | ✔ | ✔ |
| **AD19** | ✔ | ✔ | ✔ |  | **C19** | ✔ | ✔ | ✔ |
| **AD20** | ✔ | ✔ | ✔ |  | **C20** | ✔ | ✔ | ✔ |
| **AD21** | ✔ | ✔ | ✔ |  | **C21** | ✔ | ✔ | ✔ |
| **AD22** | ✔ | ✔ | ✔ |  | **C22** | ✔ | ✔ | ✔ |
| **AD23** | ✔ | ✔ | ✔ |  | **C23** | ✔ | ✔ | ✔ |
| **AD24** | ✔ | ✔ | ✔ |  | **C24** | ✔ | ✔ | ✔ |
| **AD25** | ✔ | ✔ | ✔ |  | **C25** | ✔ | ✔ | ✔ |
| **AD26** | ✔ | ✔ | ✔ |  | **C26** | ✔ | ✔ | ✔ |
|  |  |  |  |  | **C27** | ✔ | ✔ | ✔ |
|  |  |  |  |  | **C28** | ✔ | ✔ |  |

**Suppl. Table 1: Experimental sample availability of Immunohistochemical staining.**
The table summarizes the availability of DAB-stained sections for each subject across the three protein markers studied (AQP4, GFAP, Abeta42). Checkmarks indicate subjects where IHC for the respective markers was successfully performed and quantified.

| **Model: AQP4, GM, Area, n = 53, reference group = Control, R^2^ = 0.11, F = 1.53, p = 0.21** | | | | | | | |
| --- | --- | --- | --- | --- | --- | --- | --- |
| **Parameter estimates** | **Variable** | **Estimate** | **Standard error** | **Lower 95% CI** | **Upper 95% CL** | **\|t\|** | **P value** |
| β0 | Intercept | 24.5 | 19.22 | -14.15 | 63.15 | 1.274 | 0.2087 |
| β1 | Group | -3.456 | 4.076 | -11.65 | 4.739 | 0.848 | 0.4007 |
| β2 | SEX | 2.547 | 3.871 | -5.236 | 10.33 | 0.6579 | 0.5137 |
| β3 | AGE | 0.1134 | 0.2275 | -0.3439 | 0.5707 | 0.4986 | 0.6204 |
| β4 | PMI | -0.1803 | 0.1088 | -0.399 | 0.03835 | 1.658 | 0.1038 |
| **Model: AQP4, GM, Intensity, n = 52, reference group = Control, R^2^ = 0.15, F = 2.03, p = 0.11** | | | | | | | |
| **Parameter estimates** | **Variable** | **Estimate** | **Standard error** | **Lower 95% CI** | **Upper 95% CL** | **\|t\|** | **P value** |
| β0 | Intercept | 0.5212 | 0.2938 | -0.06983 | 1.112 | 1.774 | 0.0825 |
| β1 | Group | 0.04377 | 0.05598 | -0.06884 | 0.1564 | 0.782 | 0.4382 |
| β2 | SEX | 0.03208 | 0.05459 | -0.07773 | 0.1419 | 0.5877 | 0.5596 |
| β3 | AGE | 0.002801 | 0.003455 | -0.00415 | 0.009752 | 0.8106 | 0.4217 |
| β4 | PMI | -0.00384 | 0.001486 | -0.006832 | -0.0008521 | 2.585 | 0.0129 |
| **Model: AQP4, WM, Area, n = 53, reference group = Control, R^2^ = 0.16, F = 2.34, p = 0.07** | | | | | | | |
| **Parameter estimates** | **Variable** | **Estimate** | **Standard error** | **Lower 95% CI** | **Upper 95% CL** | **\|t\|** | **P value** |
| β0 | Intercept | -0.5985 | 11.97 | -24.66 | 23.46 | 0.05002 | 0.9603 |
| β1 | Group | 4.574 | 2.537 | -0.5271 | 9.674 | 1.803 | 0.0777 |
| β2 | SEX | 4.778 | 2.409 | -0.06673 | 9.622 | 1.983 | 0.0531 |
| β3 | AGE | 0.1102 | 0.1416 | -0.1744 | 0.3949 | 0.7785 | 0.4401 |
| β4 | PMI | -0.07793 | 0.0677 | -0.2141 | 0.05819 | 1.151 | 0.2554 |
| **Model: AQP4, WM, Intensity, n = 53, reference group = Control, R^2^ = 0.11, F = 1.55, p = 0.20** | | | | | | | |
| **Parameter estimates** | **Variable** | **Estimate** | **Standard error** | **Lower 95% CI** | **Upper 95% CL** | **\|t\|** | **P value** |
| β0 | Intercept | 0.4647 | 0.1106 | 0.2423 | 0.6871 | 4.201 | 0.0001 |
| β1 | Group | 0.01214 | 0.02345 | -0.03501 | 0.0593 | 0.5177 | 0.607 |
| β2 | SEX | 0.03548 | 0.02227 | -0.0093 | 0.08027 | 1.593 | 0.1177 |
| β3 | AGE | -0.00026 | 0.001309 | -0.002896 | 0.002367 | 0.202 | 0.8408 |
| β4 | PMI | -0.00109 | 0.0006259 | -0.002345 | 0.0001716 | 1.736 | 0.0889 |
| **Model: AQP4, GM, Area, n = 50, reference group = Aβ (-), R^2^ = 0.14, F = 1.80, p = 0.15** | | | | | | | |
| **Parameter estimates** | **Variable** | **Estimate** | **Standard error** | **Lower 95% CI** | **Upper 95% CL** | **\|t\|** | **P value** |
| β0 | Intercept | 21.67 | 21.02 | -20.66 | 64 | 1.031 | 0.308 |
| β1 | Group | -6.74 | 4.868 | -16.54 | 3.065 | 1.385 | 0.173 |
| β2 | SEX | 3.055 | 4.049 | -5.1 | 11.21 | 0.7544 | 0.4545 |
| β3 | AGE | 0.1819 | 0.2525 | -0.3267 | 0.6904 | 0.7203 | 0.4751 |
| β4 | PMI | -0.1803 | 0.1148 | -0.4114 | 0.05091 | 1.571 | 0.1233 |
| **Model: AQP4, GM, Intensity, n = 49, reference group = Aβ (-), R^2^ = 0.15, F = 2.00, p = 0.11** | | | | | | | |
| **Parameter estimates** | **Variable** | **Estimate** | **Standard error** | **Lower 95% CI** | **Upper 95% CL** | **\|t\|** | **P value** |
| β0 | Intercept | 0.6103 | 0.3271 | -0.04892 | 1.269 | 1.866 | 0.0687 |
| β1 | Group | -0.03269 | 0.06754 | -0.1688 | 0.1034 | 0.484 | 0.6308 |
| β2 | SEX | 0.05834 | 0.05764 | -0.05783 | 0.1745 | 1.012 | 0.317 |
| β3 | AGE | 0.002106 | 0.003867 | -0.005688 | 0.009899 | 0.5445 | 0.5888 |
| β4 | PMI | -0.00351 | 0.001566 | -0.006664 | -0.0003527 | 2.241 | 0.0301 |
| **Model: AQP4, WM, Area, n = 50, reference group = Aβ (-), R^2^ = 0.14, F = 1.77, p = 0.15** | | | | | | | |
| **Parameter estimates** | **Variable** | **Estimate** | **Standard error** | **Lower 95% CI** | **Upper 95% CL** | **\|t\|** | **P value** |
| β0 | Intercept | 0.8987 | 13.14 | -25.56 | 27.36 | 0.06841 | 0.9458 |
| β1 | Group | 2.352 | 3.043 | -3.776 | 8.48 | 0.773 | 0.4435 |
| β2 | SEX | 5.507 | 2.531 | 0.4102 | 10.6 | 2.176 | 0.0348 |
| β3 | AGE | 0.08884 | 0.1578 | -0.229 | 0.4067 | 0.563 | 0.5763 |
| β4 | PMI | -0.0502 | 0.07174 | -0.1947 | 0.09428 | 0.6998 | 0.4876 |
| **Model: AQP4, WM, Intensity, n = 50, reference group = Aβ (-), R^2^ = 0.14, F = 1.82, p = 0.14** | | | | | | | |
| **Parameter estimates** | **Variable** | **Estimate** | **Standard error** | **Lower 95% CI** | **Upper 95% CL** | **\|t\|** | **P value** |
| β0 | Intercept | 0.4493 | 0.1211 | 0.2054 | 0.6931 | 3.711 | 0.0006 |
| β1 | Group | 0.01708 | 0.02954 | -0.04241 | 0.07657 | 0.5781 | 0.5661 |
| β2 | SEX | 0.03883 | 0.02354 | -0.008595 | 0.08625 | 1.649 | 0.1061 |
| β3 | AGE | -7.9E-05 | 0.001456 | -0.003012 | 0.002853 | 0.05457 | 0.9567 |
| β4 | PMI | -0.00133 | 0.0006832 | -0.002707 | 0.0000455 | 1.948 | 0.0577 |
| **Model: GFAP, GM, Area, n = 52, reference group = Control, R^2^ = 0.16, F = 2.28, p = 0.07** | | | | | | | |
| **Parameter estimates** | **Variable** | **Estimate** | **Standard error** | **Lower 95% CI** | **Upper 95% CL** | **\|t\|** | **P value** |
| β0 | Intercept | 1.036 | 0.7241 | -0.4207 | 2.493 | 1.431 | 0.1591 |
| β1 | Group | 0.1888 | 0.1566 | -0.1263 | 0.5039 | 1.205 | 0.2341 |
| β2 | SEX | 0.3847 | 0.1438 | 0.0954 | 0.674 | 2.675 | 0.0102 |
| β3 | AGE | -0.0065 | 0.008552 | -0.0237 | 0.0107 | 0.76 | 0.451 |
| β4 | PMI | -0.00421 | 0.004336 | -0.01294 | 0.004509 | 0.9717 | 0.3362 |
| **Model: GFAP, GM, Intensity, n = 49, reference group = Control, R^2^ = 0.24, F = 3.56, p = 0.01** | | | | | | | |
| **Parameter estimates** | **Variable** | **Estimate** | **Standard error** | **Lower 95% CI** | **Upper 95% CL** | **\|t\|** | **P value** |
| β0 | Intercept | -0.0551 | 0.1421 | -0.3415 | 0.2312 | 0.3878 | 0.7 |
| β1 | Group | -0.06191 | 0.02749 | -0.1173 | -0.006509 | 2.252 | 0.0294 |
| β2 | SEX | 0.04134 | 0.02543 | -0.009902 | 0.09259 | 1.626 | 0.1111 |
| β3 | AGE | -0.00254 | 0.001644 | -0.00585 | 0.0007754 | 1.544 | 0.1298 |
| β4 | PMI | -0.00068 | 0.00076 | -0.002208 | 0.0008548 | 0.8906 | 0.378 |
| **Model: GFAP, WM, Area, n = 51, reference group = Control, R^2^ = 0.21, F = 3.09, p = 0.02** | | | | | | | |
| **Parameter estimates** | **Variable** | **Estimate** | **Standard error** | **Lower 95% CI** | **Upper 95% CL** | **\|t\|** | **P value** |
| β0 | Intercept | 0.7335 | 0.7405 | -0.757 | 2.224 | 0.9906 | 0.3271 |
| β1 | Group | -0.1924 | 0.1606 | -0.5157 | 0.1309 | 1.198 | 0.2371 |
| β2 | SEX | 0.4062 | 0.149 | 0.1063 | 0.7061 | 2.726 | 0.009 |
| β3 | AGE | -0.0008 | 0.008757 | -0.01843 | 0.01683 | 0.09155 | 0.9275 |
| β4 | PMI | -0.00425 | 0.00448 | -0.01327 | 0.004766 | 0.9491 | 0.3475 |
| **Model: GFAP, WM, Intensity, n = 51, reference group = Control, R^2^ = 0.23, F = 3.37, p = 0.02** | | | | | | | |
| **Parameter estimates** | **Variable** | **Estimate** | **Standard error** | **Lower 95% CI** | **Upper 95% CL** | **\|t\|** | **P value** |
| β0 | Intercept | -0.2487 | 0.1644 | -0.5797 | 0.08235 | 1.512 | 0.1373 |
| β1 | Group | -0.1096 | 0.03549 | -0.1811 | -0.0382 | 3.089 | 0.0034 |
| β2 | SEX | -0.02295 | 0.03245 | -0.08826 | 0.04237 | 0.7072 | 0.483 |
| β3 | AGE | 0.000532 | 0.00194 | -0.003373 | 0.004437 | 0.2741 | 0.7852 |
| β4 | PMI | -0.00037 | 0.0009716 | -0.002326 | 0.001586 | 0.381 | 0.705 |
| **Model: GFAP, GM, Area, n = 50, reference group = Aβ (-), R^2^ = 0.18, F = 2.45, p = 0.06** | | | | | | | |
| **Parameter estimates** | **Variable** | **Estimate** | **Standard error** | **Lower 95% CI** | **Upper 95% CL** | **\|t\|** | **P value** |
| β0 | Intercept | 1.178 | 0.7727 | -0.3784 | 2.734 | 1.524 | 0.1344 |
| β1 | Group | 0.2622 | 0.1805 | -0.1013 | 0.6257 | 1.453 | 0.1533 |
| β2 | SEX | 0.3831 | 0.1492 | 0.0825 | 0.6837 | 2.567 | 0.0137 |
| β3 | AGE | -0.00919 | 0.009242 | -0.0278 | 0.009429 | 0.9939 | 0.3256 |
| β4 | PMI | -0.00424 | 0.004224 | -0.01274 | 0.004274 | 1.002 | 0.3215 |
| **Model: GFAP, GM, Intensity, n = 47, reference group = Aβ (-), R^2^ = 0.16, F = 1.96, p = 0.12** | | | | | | | |
| **Parameter estimates** | **Variable** | **Estimate** | **Standard error** | **Lower 95% CI** | **Upper 95% CL** | **\|t\|** | **P value** |
| β0 | Intercept | -0.09849 | 0.161 | -0.4234 | 0.2264 | 0.6117 | 0.544 |
| β1 | Group | -0.03626 | 0.03352 | -0.1039 | 0.03139 | 1.082 | 0.2856 |
| β2 | SEX | 0.04248 | 0.02773 | -0.01347 | 0.09844 | 1.532 | 0.133 |
| β3 | AGE | -0.00199 | 0.001872 | -0.005762 | 0.001792 | 1.06 | 0.295 |
| β4 | PMI | -0.00102 | 0.0007924 | -0.002618 | 0.0005798 | 1.286 | 0.2054 |
| **Model: GFAP, WM, Area, n = 49, reference group = Aβ (-), R^2^ = 0.19, F = 2.59, p = 0.05** | | | | | | | |
| **Parameter estimates** | **Variable** | **Estimate** | **Standard error** | **Lower 95% CI** | **Upper 95% CL** | **\|t\|** | **P value** |
| β0 | Intercept | 0.3515 | 0.7951 | -1.251 | 1.954 | 0.442 | 0.6607 |
| β1 | Group | -0.1223 | 0.1857 | -0.4965 | 0.252 | 0.6584 | 0.5137 |
| β2 | SEX | 0.4093 | 0.1556 | 0.09574 | 0.7229 | 2.631 | 0.0117 |
| β3 | AGE | 0.004054 | 0.009524 | -0.01514 | 0.02325 | 0.4257 | 0.6724 |
| β4 | PMI | -0.00559 | 0.00441 | -0.01448 | 0.003293 | 1.269 | 0.2113 |
| **Model: GFAP, WM, Intensity, n = 49, reference group = Aβ (-), R^2^ = 0.19, F = 6.66, p = 0.04** | | | | | | | |
| **Parameter estimates** | **Variable** | **Estimate** | **Standard error** | **Lower 95% CI** | **Upper 95% CL** | **\|t\|** | **P value** |
| β0 | Intercept | -0.3313 | 0.1822 | -0.6984 | 0.03582 | 1.819 | 0.0758 |
| β1 | Group | -0.109 | 0.04167 | -0.193 | -0.02503 | 2.616 | 0.0121 |
| β2 | SEX | -0.0116 | 0.03463 | -0.0814 | 0.0582 | 0.3349 | 0.7393 |
| β3 | AGE | 0.001939 | 0.002179 | -0.002452 | 0.006331 | 0.89 | 0.3783 |
| β4 | PMI | -0.00089 | 0.0009699 | -0.002846 | 0.001064 | 0.9185 | 0.3634 |

**Suppl. Table 2: Multiple linear regression models for AQP4 and GFAP expression.**
Each block of the table reports one linear model separately for each marker (AQP4, GFAP), tissue region (GM: gray matter, WM: white matter), and metric (Area %, Mean Intensity). For each model, the sample size (n), the reference group, the coefficient of determination (R^2^), and the overall model F-statistic with its p-value are reported. Within each model, rows report the parameter estimates: β0 (intercept), β1 (Group), β2 (Sex), β3 (Age), and β4 (PMI). For each parameter, the estimate value, the standard error, the lower and upper bounds of the 95% confidence interval, absolute t-statistic (|t|), and p-value are shown.
